# Treatment of Multidrug-Resistant Leukemia Cells by Novel Artemisinin-, Egonol-, and Thymoquinone-Derived Hybrid Compounds

**DOI:** 10.3390/molecules23040841

**Published:** 2018-04-06

**Authors:** Lisa Gruber, Sara Abdelfatah, Tony Fröhlich, Christoph Reiter, Volker Klein, Svetlana B. Tsogoeva, Thomas Efferth

**Affiliations:** 1 Department of Pharmaceutical Biology, Institute of Pharmacy and Biochemistry, Johannes Gutenberg University, 55128 Mainz, Germany; gruber.lisa@googlemail.com (L.G.); saabdelf@uni-mainz.de (S.A.); 2 Department of Chemistry and Pharmacy, Organic Chemistry Chair I and Interdisciplinary Center for Molecular Materials (ICMM), University of Erlangen-Nuremberg, 91058 Erlangen, Germany; tony.froehlich@fau.de (T.F.); christoph.reiter@fau.de (C.R.); volker.klein@fau.de (V.K.); svetlana.tsogoeva@fau.de (S.B.T.)

**Keywords:** chemotherapy, multi-drug resistance, artemisinin, egonol, thymoquinone, hybrids

## Abstract

Two major obstacles for successful cancer treatment are the toxicity of cytostatics and the development of drug resistance in cancer cells during chemotherapy. Acquired or intrinsic drug resistance is responsible for almost 90% of treatment failure. For this reason, there is an urgent need for new anticancer drugs with improved efficacy against cancer cells, and with less toxicity on normal cells. There are impressive examples demonstrating the success of natural plant compounds to fight cancer, such as *Vinca* alkaloids, taxanes, and anthracyclines. Artesunic acid (ARTA), a drug for malaria treatment, also exerts cytotoxic activity towards cancer cells. Multidrug resistance often results from drug efflux pumps (ABC-transporters) that reduce intracellular drug levels. Hence, it would be interesting to know, whether ARTA could overcome drug resistance of tumor cells, and in what way ABC-transporters are involved. Different derivatives showing improved features concerning cytotoxicity and pharmacokinetic behavior have been developed. Considering both drug sensitivity and resistance, we chose a sensitive and a doxorubicin-resistant leukemia cell line and determined the killing effect of ARTA on these cells. Molecular docking and doxorubicin efflux assays were performed to investigate the interaction of the derivatives with *P*-glycoprotein. Using single-cell gel electrophoresis (alkaline comet assay), we showed that the derivatives of ARTA induce DNA breakage and accordingly programmed cell death, which represents a promising strategy in cancer treatment. ARTA activated apoptosis in cancer cells by the iron-mediated generation of reactive oxygen species (ROS). In conclusion, ARTA derivatives may bear the potential to be further developed as anticancer drugs.

## 1. Introduction

The active compound artemisinin is derived from *Artemisia annua* L. (*qinhao, sweet wormwood*), a medical plant used in traditional Chinese medicine, and its semisynthetic derivatives artesunate and dihydroartemisinin exert not only antimalarial activity, even to otherwise drug-resistant *Plasmodia* [1], but also display inhibitory activity towards other diseases, including cancer in vitro [2,3,4,5,6,7,8] and in vivo [9,10]. Artemisinin and its derivatives also revealed anticancer activity in clinical pilot trials with human and veterinarian cancer patients [11,12,13,14,15,16,17,18]. More recently, it turned out that the bioactivity spectrum is much broader, and that artemisinin and its derivatives may also be valuable to treat other diseases, e.g., viral infections, schistosomiasis, trypanosomiasis, atherosclerosis, or diabetes [19,20,21,22,23]. Interesting features of artemisinin include activity against multidrug-resistant cancer cells [24], and good tolerance [25]. Because artemisinin has saved the lives of millions of patients, the Chinese scientist Youyou Tu, who discovered the antimalarial activity of this compound found in *A. annua*, was honored with the Nobel Prize for Medicine or Physiology in 2015 [26]. An endoperoxide bridge constitutes the active moiety, because its cleavage leads to the formation of reactive oxygen species (ROS) [27] and carbon-centered radicals [28]. In the malaria parasites, artesunic acid (ARTA) causes the iron(II)-mediated alkylation of heme and several other proteins, such as the translationally controlled tumor protein (TCTP), histidine-rich protein, and the sarco/endoplasmic reticulum Ca^2+^ ATPase (SERCA) [29,30]. Tumor cells contain less iron than erythrocytes, but more than other normal tissues [31]. Iron may also be critical for the action of artemisinin-type drugs towards tumor cells, because it is correlated with the expression of the transferrin receptor (CD71), which is responsible for cellular iron uptake [32,33,34,35,36,37].

In malaria treatment, there is no cross-resistance of artemisinin and its derivatives to other antimalarial drugs. Therefore, it is reasonable to ask, whether or not ARTA is involved in the multidrug-resistance (MDR) phenotype in tumor cells.

ABC (ATP-binding cassette) transporters have a key function in MDR, where tumor cells develop resistance to a relatively wide range of drugs that have no structural or pharmacological similarities [38]. P-glycoprotein is a well-known member of this family and has a diverse spectrum of substrates, varying in size from 200 to 1900 Da [39]. It is responsible for the efflux of many xenobiotics, including numerous anticancer agents. In 1981, Tsuruo et al. first described verapamil, a calcium channel blocker, as an inhibitor of P-glycoprotein-mediated drug efflux [40]. Since then, there is still an ongoing search for new modulators of P-glycoprotein [41,42,43,44,45,46,47,48,49,50,51,52,53]. First-generation modulators, originally not used in cancer therapy, were highly toxic at suitable doses. Their derivatives, the second-generation modulators, had better efficacies, but had alarming pharmacokinetic interactions with anticancer drugs. Hence, new third-generation drugs have been developed with the hope that they would have a higher specificity for P-glycoprotein and fewer toxic effects. Three major binding sites were described for P-glycoprotein [54]: the H-site favors Hoechst 33,342, the R-site interacts with rhodamine 123, and a third site named the M-(modulatory) site.

To investigate the interaction with P-glycoprotein, we screened new ARTA derivatives, the design and development of which was inspired by the powerful concepts of natural product hybridization [55,56] and dimerization [57]. In addition, the possible mode of binding of these compounds was studied using molecular docking. Furthermore, we chose sensitive and doxorubicin-resistant leukemia cell lines to determine the cytotoxicity of a panel of ARTA derivatives on these cells. ARTA induced DNA damage and triggered apoptotic cell death in various tumor cell lines [24,58,59,60,61]. Therefore, we quantified the DNA damage of this series of ARTA derivatives by the alkaline comet assay.

## 2. Materials and Methods

### 2.1. Compounds

**Scheme 1:**
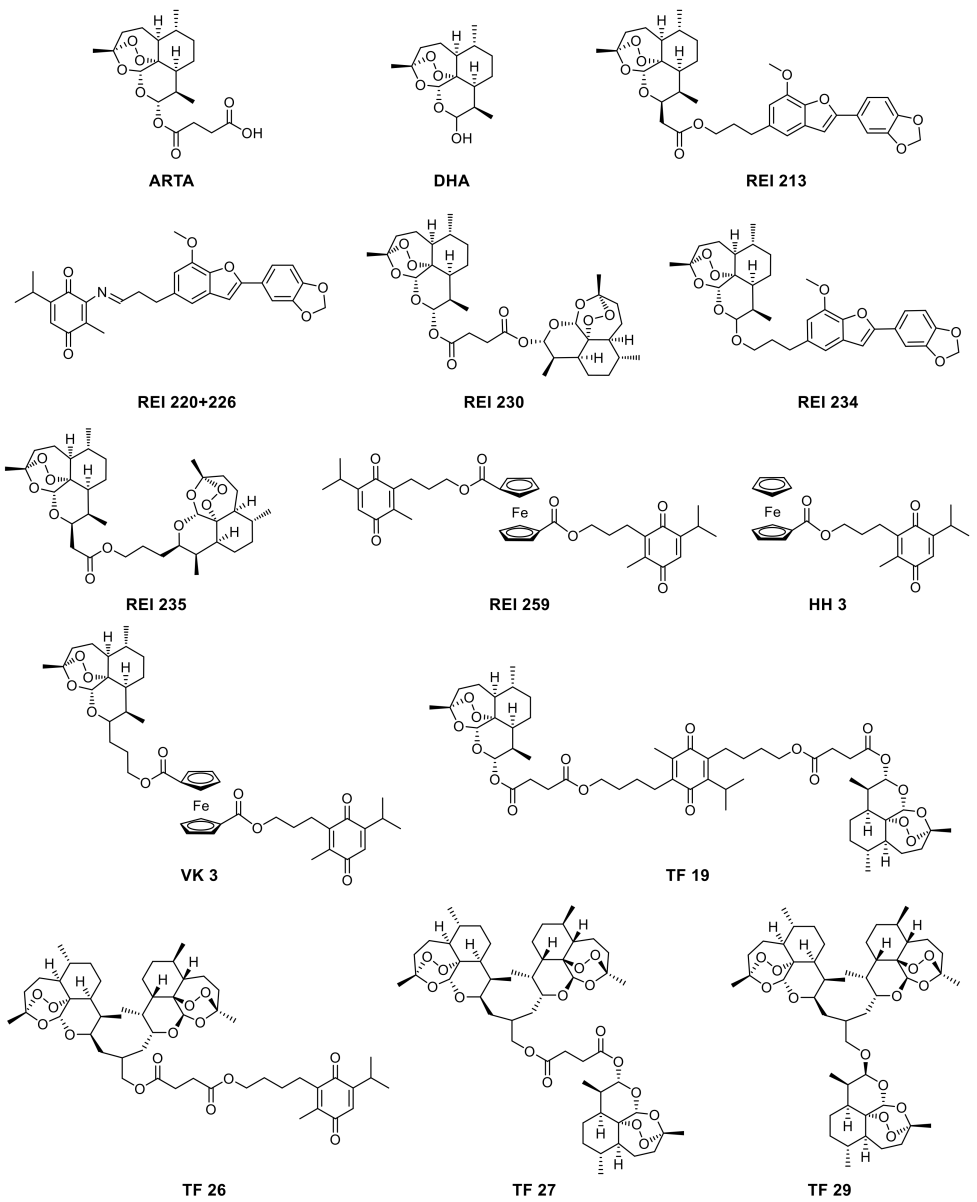
Chemical structures of artemisinin-, egonol-, and thymoquinone-based hybrids, dimers, and trimers studied in this work.

The syntheses of compounds REI230, REI235, TF27, and TF29 have been previously reported, [62] and are shown in Scheme 1. The chemical structures and physical–chemical characterization of compounds TF19, TF26, REI213, REI220+226, REI234, REI259, VK3, and HH3 are presented in the Supplementary Materials.

### 2.2. Cell Culture

Human leukemic CCRF-CEM and P-glycoprotein-expressing CEM/ADR5000 cells were obtained from the University of Jena (Department for Pediatrics, University of Jena, Jena, Germany). Cells were cultured in RPMI 1640 medium (Life Technologies, Schwerte, Germany), supplemented with 10% FBS (Life Technologies) and 1% penicillin (1000 U/mL)/streptomycin (100 µg/mL) (Life Technologies). They were maintained in a humidified, 5% CO_2_ supplied atmosphere in an incubator at 37 °C. CEM/ADR5000 cells were treated with 5000 ng/mL doxorubicin (Medical Center, Johannes Gutenberg University, Mainz, Germany) once per week to retain their resistance phenotype. The MDR profile has been previously reported [63,64,65]. Cells were passaged twice a week and then used for experiments in the logarithmic phase.

### 2.3. Resazurin Reduction Assay

The resazurin reduction assay was used to detect the cytotoxicity of ARTA derivatives towards sensitive and multidrug-resistant cells. Viable cells are able to reduce resazurin to the highly fluorescent resorufin, and this fluorescence can be measured with the Tecan Reader Infinite m200 Pro (Crailsheim, Germany). The method was previously described [66,67]. Briefly, CCRF-CEM and CEM/ADR5000 cells were seeded in the appropriate density (10,000 cells/well) in 96-well cell plates, with a total volume of 200 µL per well. Compounds were added in varying concentrations (0.001, 0.003, 0.01, 0.03, 0.1, 0.3, 1, 3, 10, and 100 µM). Each concentration was tested six times per experiment, and each experiment was repeated three times. Additionally, we tested the CEM/ADR5000 cells with doxorubicin alone and in combination with three different ARTA derivatives (10 µM) or verapamil (0.1, 0.3, 1, 3, 10, and 100 µM). After 72 h at 37 °C and 5% CO_2_, 20 µL resazurin (Sigma-Aldrich, Taufkirchen, Germany) 0.01% *w*/*v* in ddH_2_O was added to each well and further incubated for 4 h. The plates were measured using an excitation wavelength of 544 nm and an emission wavelength of 590 nm. The test compound concentrations required to inhibit 50% of cell proliferation were represented by IC_50_ values calculated from dose–response curves.

### 2.4. Molecular Docking

Two-dimensional structures of ARTA and its derivatives were drawn and converted to 3D structures using the Corina Online Demo, and were saved in PDB format. Using the X-ray crystallography-based structure of a mouse P-glycoprotein as a template (PDB code: 5KOY), the homology structure of human P-glycoprotein was modeled as described [68]. The PDB file was converted to the PDBQT format using AutodockTools-1.5.6rc3. A grid box (coordinates of three dimensions: [grid center]: X: 21.092, Y: 92.594 and Z: 24.0; number of grid points in the three dimensions [npts]: X: 120, Y: 98 and Z: 100; spacing: 0.375) was constructed to define the transmembrane docking spaces of every type of atom in the ligand energies, which are used to predict the binding energies of the ligand; transmembrane docking spaces were calculated with the Autogrid 4.2 (The Scripps research Institute, Molecular Graphics Laboratory, La Jolla, CA, USA). Docking parameters were set to 250 runs and a 2,500,000 energy evaluation was set for each cycle. Using the Autodock 4.2 (Molecular Graphics Laboratory), we docked every ligand via the Lamarckian algorithm. The binding energies and interacting amino acids were received from DLG files, and the images were obtained using Visual Molecular Dynamics VMD (University of Illinois at Urbana Champaign, Champaign, IL, USA).

### 2.5. Flow Cytometry

CCRF-CEM and CEM/ADR5000 cells were exposed to doxorubicin (10 µM) (in the presence and absence of verapamil) and ARTA and its derivatives (10 µM). After incubation for 24 h, cells were harvested by centrifugation at 1500× *g* for 5 min. The supernatant was removed and the cells were suspended in a RPMI colorless medium. The fluorescence intensity of the intracellular doxorubicin was determined using a flow cytometer FACScalibur (Becton-Dickinson, Heidelberg, Germany), equipped with an ultraviolet argon laser (excitation at 488 nm, emission at 530/30 and 570/30 nm band-pass filters). The experiment was repeated thrice. Viable cells were gated, and we obtained the log fluorescence of single cells in forward and side light-scatter, based on the acquisition of data from 20,000 cells.

### 2.6. Single Cell Gel Electrophoresis (Alkaline Comet Assay)

DNA single-strand breaks were determined and calculated by single-cell gel electrophoresis. We used the OxiSelect™ Comet Assay Kit (Cell Biolabs-BIOCAT, Heidelberg, Germany). The alkaline comet assay detects both single and double DNA strand breaks. Radical molecules formed by ARTA derivatives generate DNA lesions and strand breaks. The DNA fragments migrate in the electrophoretic gel and form comets. With increasing DNA damage, the tail intensity increases rather than its length; tail length is determined primarily by the length of the loops. Here, we used a recently described protocol [69]. Exponentially growing cells were exposed to ARTA derivatives (10 µM) for 24 h, and then processed according to the manufacturer’s instructions. We varied both lysis times and electrophoresis times by 30 min. Fifteen cells per sample were analyzed with casplab 1.1.3b.2 software (http://casplab.com), (version 1.1.3 beta 2, CASPLAB, Wroclaw, Poland).

## 3. Results

To analyze the cytotoxic effects of ARTA derivatives, CCRF-CEM and P-glycoprotein-overexpressing CEM/ADR5000 cells were treated with the compounds for 72 h, and then the cell viability was evaluated using the resazurin reduction assay. Since drug resistance is a major problem in cancer chemotherapy, we assessed whether or not the derivatives bypassed MDR. The results are shown in Table 1. Most of the derivatives inhibited proliferation by 50% at concentrations below 10 µM. With the exception of TF26 and TF29, CEM/ADR5000 cells showed no or negligible cross-resistance towards the derivatives. Except for TF26 and TF29, the degree of resistance of the CEM/ADR5000 cells was much lower when compared to doxorubicin.

**Figure 1:**
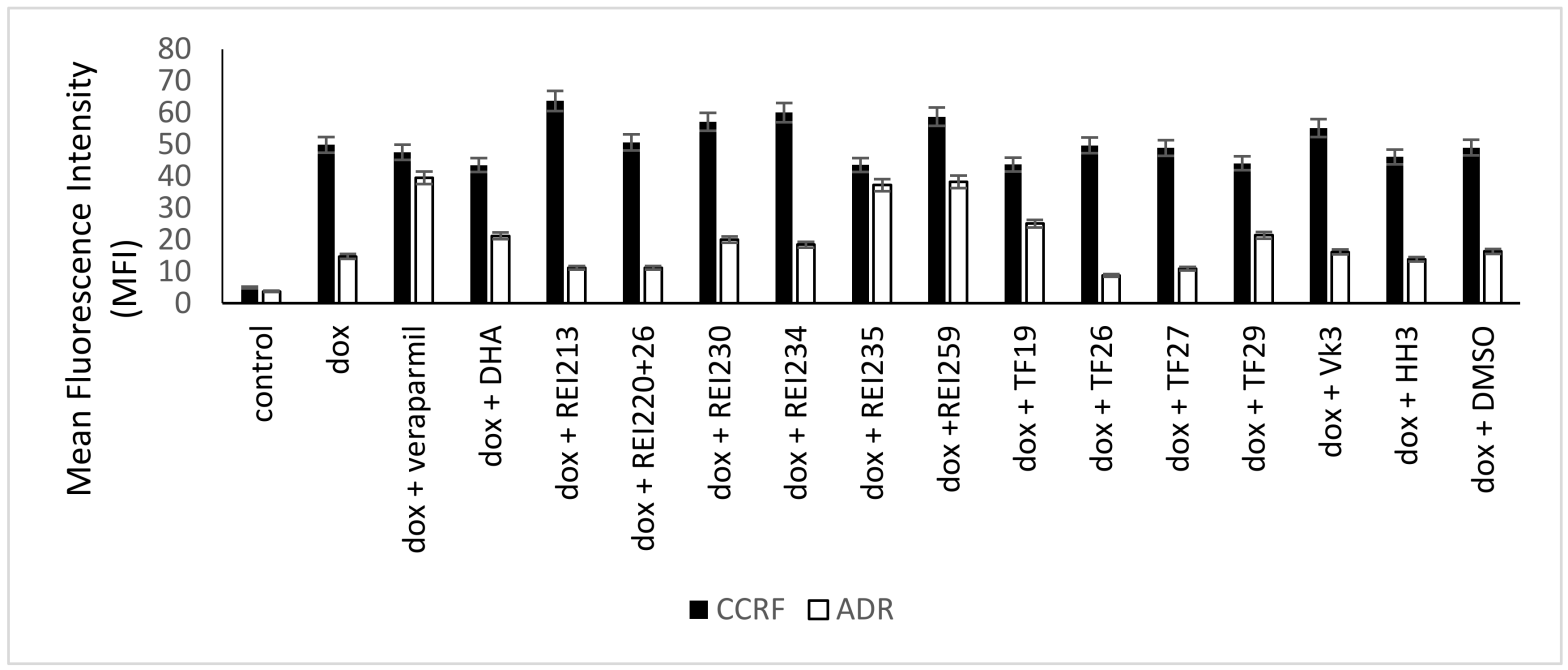
Flow cytometric quantification of intracellular doxorubicin fluorescence in CCRF-CEM and CEM/ADR5000 cells treated with doxorubicin alone, doxorubicin and artemisinin-, egonol-, and thymoquinone-based hybrids, or doxorubicin and the P-glycoprotein inhibitor verapamil, as a positive control after incubation for 24 h.

To measure the P-glycoprotein inhibitory activity of the ARTA derivatives, doxorubicin efflux assays were performed; relative intracellular doxorubicin concentrations were measured by flow cytometry. For comparison, the doxorubicin efflux assays were executed in the presence of a known P-glycoprotein modulator, verapamil. Figure 1 shows the results of the flow cytometric measurements. We measured low fluorescence intensities (MFIs). The MFIs of doxorubicin-treated cells were the lowest because of the efflux transport by P-glycoprotein. Concurrent treatment with verapamil inhibited P-glycoprotein, hence, these MFI values were the highest. Treatment with doxorubicin and the ARTA derivatives was found to be between both standards. Some of the derivatives did not interact with P-glycoprotein, and some inhibited the ABC-transporter (e.g., REI235, REI259, and TF19).

To confirm the flow cytometry results, we carried out resazurin reduction assays by combining doxorubicin with the P-glycoprotein-modulating ARTA derivatives. We tested doxorubicin alone, in combination with verapamil (a known modulator of P-glycoprotein), and each of the three ARTA derivatives. Table 2 summarizes the IC_50_ values and the degrees of resistance reversal, which were calculated by dividing the IC_50_ values of doxorubicin alone by the IC_50_ values of doxorubicin in combination with the modulators.

To consider the mode of binding of the ARTA derivatives, we performed molecular docking on human P-glycoprotein at the transmembrane domain (TDM). Table 3 summarizes the results for each compound, providing the lowest binding energies. The total number of interacting amino acids and the amino acids involved in hydrogen bonds are also displayed in Table 3.

**Figure 2:**
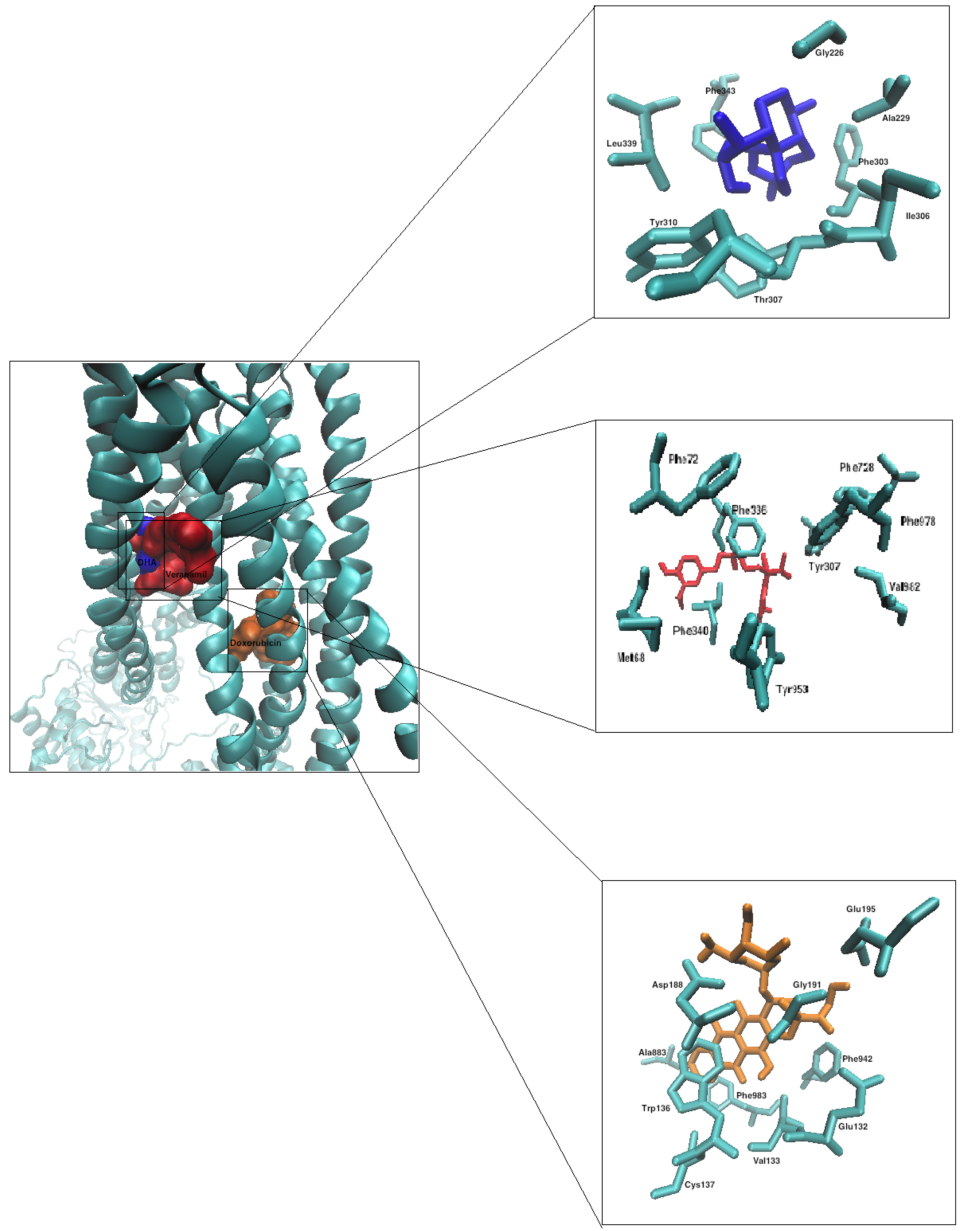
Molecular Docking of dihydroartemisinin (blue), verapamil (red), and doxorubicin (orange) to P-glycoprotein (represented in a new cartoon style shown as cyan), corresponding interacting amino acids are shown for each compound.

Figure 2 demonstrates the binding modes of dihydroartemisinin, verapamil, and doxorubicin with the respective interacting amino acids.

**Figure 3:**
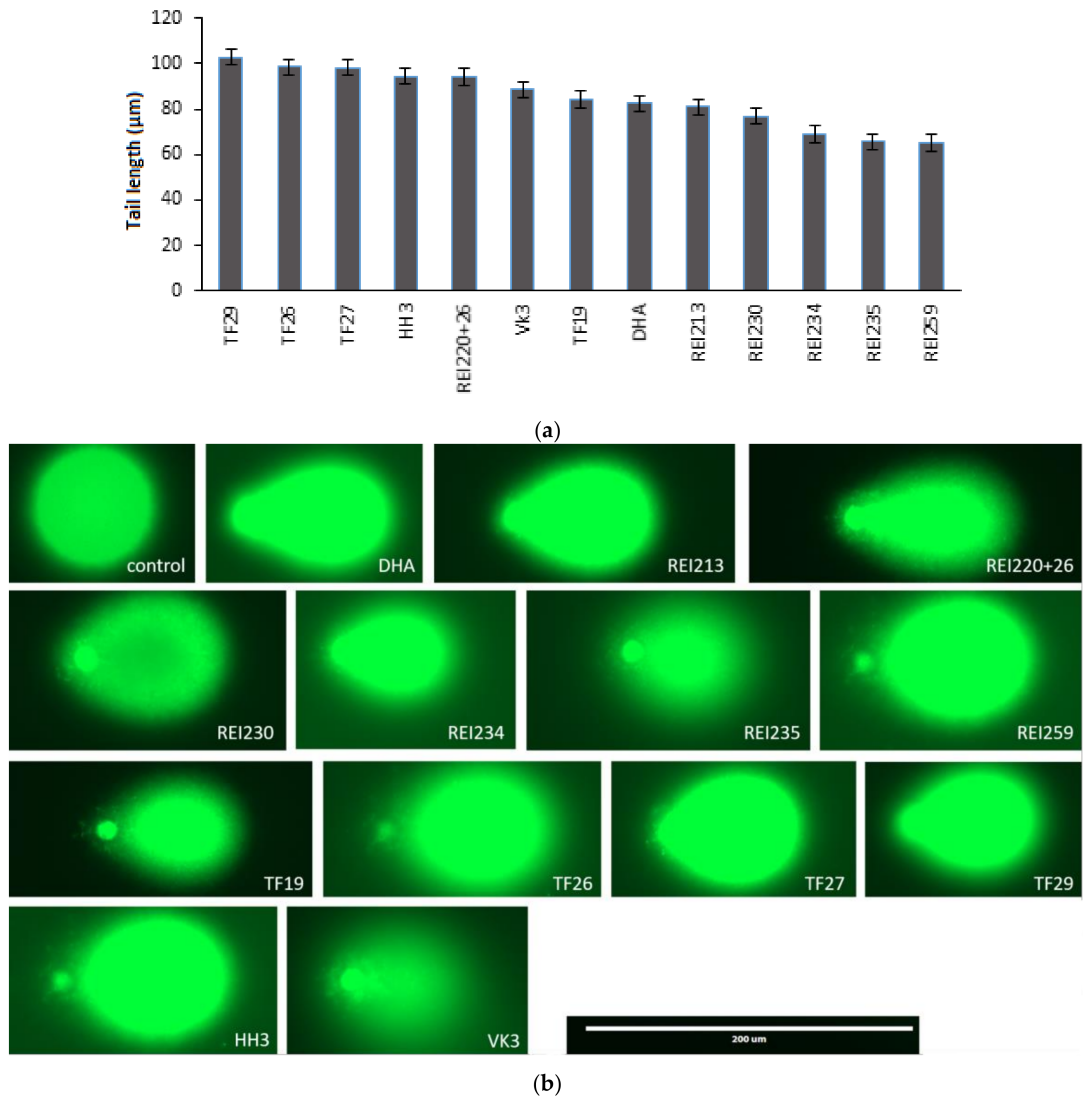
DNA damage induced by artemisinin-, egonol-, and thymoquinone-based hybrids. CCRF-CEM cells were each treated with10 µM of the above compounds and harvested for 24 h. The cells were subjected to alkaline single-cell gel electrophoresis as described. (**a**) Quantified tail lengths are the mean values of at least three independent experiments ± SD; (**b**) Representative images of cells treated with ARTA derivatives.

Furthermore, we examined the induction of DNA damage triggered by the artemisinin-, egonol-, and thymoquinone-based hybrids. For this reason, we performed the alkaline comet assay. CCRF-CEM cells were incubated with the derivatives for 24 h. As shown in Figure 3a, the tail lengths varied between 102.91 (± 0.53) and 65.15 (± 0.20) µm. Representative images of CCRF-CEM cells subjected to the alkaline comet assay upon treatment with ARTA derivatives are shown in Figure 3b.

## 4. Discussion

In the present investigation, we demonstrated the cytotoxic activity of 12 novel ARTA-based derivatives on sensitive CCRF-CEM and multidrug-resistant CEM/ADR5000 cells. The IC_50_ values ranged from 0.0018 to 43.685 µM, and for most of the compounds we achieved low resistance indices. It is known for ARTA and its derivatives that their cytotoxicity is attributed to the cleavage of the endoperoxide bridge and subsequent ROS generation. One of the compounds, REI220+26, displayed higher cytotoxicity in CEM/ADR5000 than in CCRF-CEM cells. This ability of compounds to kill multidrug-resistant cells with greater efficacy than their parenteral drug-sensitive cells is termed collateral sensitivity [70,71]. The mechanism is still not completely understood, but it has been proposed that substances discharged by the ABC transporter deplete ATP [72], subsequent replenishment of ATP generates ROS by oxidative phosphorylation [73], and many collateral sensitive substances that are lipophilic re-enter the cell. Hence, a futile cycling [74] may develop, leading to ATP depletion and preferential killing of multidrug-resistant cells. Previously, it has been shown that twice the amount of ATP is consumed in multidrug-resistant cell lines in comparison to sensitive cells [74]. CEM/ADR5000 cells overexpress P-glycoprotein [63,65,75] and exhibit MDR with cross-resistance between anthracyclines, *Vinca* alkaloids, taxanes, and other drugs [64]. However, treatment of the CEM/ADR5000 cells with ARTA derivatives resulted in considerably enhanced intracellular retention of doxorubicin through inhibition of P-glycoprotein activity. The results obtained by flow cytometry were confirmed by resazurin assays that assessed the effect of the three most potent compounds REI235, REI259, and TF19 in combination with doxorubicin.

Furthermore, we used in silico molecular docking to investigate the possible interactions of these compounds with P-glycoprotein. With the exception of TF19, all derivatives revealed a similar binding site at the transmembrane region of P-glycoprotein. This binding site was close to the one of verapamil, and similar degrees of resistance reversal were observed. For this reason, we conclude that both the ARTA derivatives and verapamil may share a similar mechanism of inhibiting P-glycoprotein. Verapamil inhibits P-glycoprotein-mediated drug efflux in a competitive manner.

Most anti-cancer drugs kill tumor cells by the induction of programmed cell death, and it has been first shown by Efferth et al. that ARTA induces apoptosis in leukemia cells [4]. This result was subsequently corroborated by several other groups (for review see [6]). ARTA induces apoptosis in a different manner than doxorubicin. This may be the reason why ARTA induced apoptosis in doxorubicin-resistant cells [76]. In this study, we demonstrated by comet assays that the ARTA derivatives caused DNA damage. Recently, it was shown that ARTA induced DNA damage, presumably by ROS [59]. Furthermore, protein alkylation is a reason for ARTA-induced cytotoxicity [77,78]. The cleavage of the endoperoxide moiety of ARTA in the presence of ferrous iron by a Fenton-type reaction leads to the formation of ROS, as well as carbon-centered radical molecules. In conclusion, we described a panel of novel ARTA derivatives, most of which were not cross-resistant or collateral sensitive in multidrug-resistant cells. Some of the derivatives inhibited doxorubicin efflux by P-glycoprotein, leading to the reversal of doxorubicin resistance. Our data might inspire further investigations to clarify the exact signaling pathways of ARTA in cancer cells. ARTA derivatives may be useful drugs to fight MDR in cancer cells and improve the treatment of refractory tumors in clinics.

## Figures and Tables

**Table 1:** The cytotoxicity of artesunic acid (ARTA) derivatives and doxorubicin. CCRF-CEM and P-glycoprotein-expressing CEM/ADR5000 cells were incubated for 72 h with artemisinin-, egonol-, and thymoquinone-based hybrids. Resazurin assays were performed to determine dose–response curves and IC_50_ measurements were calculated (mean ± SD). Experiments were repeated at least twice. Resistance indices were obtained by dividing the IC_50_ values on the resistant cell line through that of the sensitive cell line.

Compound	IC_50_ (µM) ± SD		Degree of Resistance
	CCRF-CEM	CEM/ADR5000	
Doxorubicin	0.0033 ± 0.00065	1.613 ± 0.166	488.79
Artesunic acid	0.069 ± 0.03	0.189 ± 0.003	2.739
DHA	0.085 ± 0.003	0.265 ± 0.008	3.118
REI213	0.568 ± 0.215	0.582 ±0.224	1.025
REI220+26	43.685 ± 4.385	17.450 ± 1.010	0.399
REI230	2.748 ± 0.021	2.789 ± 0.018	1.015
REI234	0.0018 ± 0.0001	0.0068 ± 0.0006	3.778
REI235	0.092 ± 0.006	0.199 ± 0.023	2.163
REI259	0.876 ± 0.192	3.852 ± 1.021	4.397
TF19	6.071 ± 0.247	5.663 ± 0.190	0.933
TF26	0.0027 ± 0.001	7.872 ± 0.594	2915.556
TF27	0.0024 ± 0.0001	0.196 ± 0.008	81.667
TF29	0.0021 ± 0.0003	0.485 ± 0.210	230.952
HH3	7.343 ± 0.911	7.071 ± 1.408	0.963
VK3	0.134 ± 0.140	5.210 ± 0.153	38.881

**Table 2:** IC_50_ values of CEM/ADR5000 upon treatment of doxorubicin combined with verapamil, or three hybrids, as measured by resazurin assays and the degrees of resistance reversal. Verapamil (20 µM) or derivatives (10 µM) were used in combination with doxorubicin (0.1–100 µM). Experiments were repeated at least twice, and for each concentration at least in triplicate.

Modulator	IC_50_ (µM) in Combination with Doxorubicin ± SD	Fold Change in IC_50_ (Degree of Resistance Reversal)
-	2.19 ± 0.041	-
Verapamil	0.69 ± 0.170	3.17
REI259	0.84 ± 0.020	2.61
REI235	0.85 ± 0.077	2.58
TF19	1.43 ± 0.034	1.53

**Table 3:** Molecular docking of artemisinin-, egonol-, and thymoquinone-based hybrids on homology-modelled human P-glycoprotein in the transmembrane domain.

Compound	Lowest Binding Energy (kcal/mol)	Mean Binding Energy (Kcal/mol)	Number of Interacting AA	AA Involved in H-Bond	pKi
REI213	−12.945 (± 0.015)	−11.83 (± 0.11)	13	Gln725, Tyr953	9.4
REI20+26	−11.055 (± 0.105)	−10.31 (± 0.0)	13	-	7.8
REI230	−12–78 (± 0.01)	12.34 (± 0.12)	11	-	9.3
REI234	−12.84 (± 0.035)	12.185 (± 0.095)	13	Tyr953	9.5
REI235	−13.01 (± 0.01)	12.24 (± 0.0)	13	-	9.5
TF19	11.425 (± 0.755)	11.015 (± 0.345)	17	-	8.9
TF26	−13.68 (± 0.425)	13.3 (± 0.81)	15	Gln195	9.7
TF27	−14.815 (± 0.205)	−12.75 (± 0.37)	15	Gln990	11.0
TF29	−15.545 (± 0.049)	−13.845 (± 0.665)	10	Gln990	11.75

## Supplementary Materials

The following are available online. Physical-chemical characterization of selected compounds and representative flow cytometric histograms of doxorubicin uptake assay in CEM/ADR5000 cells.

## Abbreviations

AA, amino acid; ABC, ATP-binding cassette; ARTA, artesunic acid; DOX, doxorubicin; MDR, multidrug resistance; ROS, reactive oxygen species; SERCA, sarco/endoplasmic reticulum Ca^2+^ATPase; TCTP, translationally controlled tumor protein.
