# Supplementary material for: Treatment of Multidrug-Resistant Leukemia Cells by Novel Artemisinin-, Egonol-, and Thymoquinone-Derived Hybrid Compounds
Source: Molecules. 2018 Apr 6;23(4):841. doi: 10.3390/molecules23040841 (PMC6017613; doi:10.3390/molecules23040841)
Supplement: Supplementary file 1 [file molecules-23-00841-s001.pdf]

Supporting Information

Treatment of Multidrug-Resistant Leukemia Cells by Novel Artemisinin-, Egonol-, and Thymoquinone-Derived Hybrid Compounds

Lisa Gruber[1], Sara Abdelfatah[1], Tony Fröhlich[2], Christoph Reiter[2], Volker Klein[2], Svetlana B. Tsogoeva[2], Thomas Efferth[1]*

[1] Department of Pharmaceutical Biology, Institute of Pharmacy and Biochemistry, Johannes Gutenberg University, Mainz, Germany
[2] Department of Chemistry and Pharmacy, Organic Chemistry Chair I and Interdisciplinary Center for Molecular Materials (ICMM), University of Erlangen-Nuremberg, Erlangen, Germany

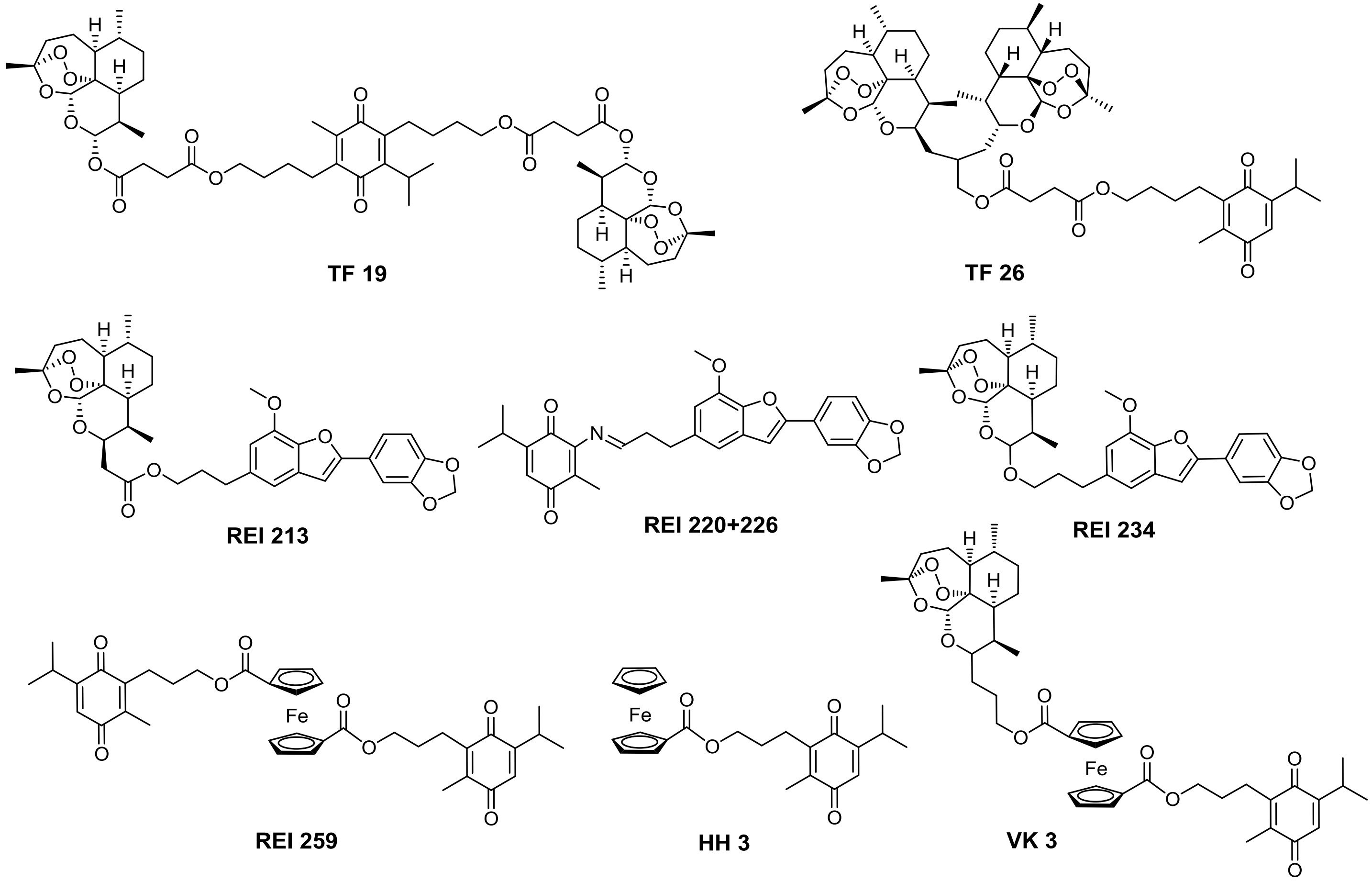

Figure S1: Chemical structures of new artemisinin-based hybrids, dimers and trimers studied in this work.

Purity Data

TF 19: MS (ESI): *m/z* = 1064 $[M+Na]^+$. HRMS (ESI): *m/z* calcd. for $[C_{56}H_{80}NaO_{18}]^+$: 1063.5237, found: 1063.5244. Anal. calcd. for $C_{56}H_{80}O_{18}$: C, 64.60; H, 7.74; Found: C, 64.58; H, 7.84.

TF 26: MS (ESI): *m/z* = 948 $[M+Na]^+$. HRMS (ESI): *m/z* calcd. for $[C_{52}H_{76}NaO_{14}]^+$: 947.5127, found: 947.5151. Anal. calcd. for $C_{52}H_{76}O_{14}$: C, 67.51; H, 8.28; Found: C, 67.34; H, 8.34.

REI 213: MS (ESI): *m/z* = 657 $[M+Na]^+$. HRMS (ESI): *m/z* calcd. for $[C_{36}H_{42}NaO_{10}]^+$ 657.2670, found 657.2682. Anal. calcd. for $C_{36}H_{42}O_{10}$: C, 68.12; H, 6.67; Found: C, 68.20; H, 7.02.

REI 220+226: MS (MALDI-TOF, without matrix): *m/z* = 485 $[M]^+$. HRMS (ESI): *m/z* calcd. for $[C_{29}H_{28}NO_6]^+$ 486.1911, found 486.1897. Anal. calcd. for $C_{29}H_{27}NO_6$: C, 71.74; H, 5.61; N, 2.88; Found: C, 71.39; H, 5.57; N, 2.81.

REI 234: MS (MALDI-TOF, without matrix): *m/z* = 592 $[M]^+$. HRMS (ESI): *m/z* calcd. for $[C_{34}H_{40}NaO_9]^+$ 615.2565, found 615.2588. Anal. calcd. for $C_{34}H_{40}O_9$**:** C, 68.90; H, 6.80; Found: C, 68.99; H, 6.97.

REI 259: MS (ESI): *m/z* = 705 $[M+Na]^+$, 1387 $[2M+Na]^+$. HRMS (ESI): *m/z* calcd. for $[C_{38}H_{42}FeNaO_8]^+$ 705.2122, found 705.2128. Anal. calcd. for $C_{38}H_{42}FeO_8$: C, 66.86; H, 6.20; Found: C, 66.86; H, 6.07; N, 0.08.

HH 3: MS (ESI): *m/z* = 434 [M]. Anal. calcd. for: $[C_{24}H_{26}FeO_4]$: C, 66.37; H, 6.03. Found: C, 66.70; H, 6.23.

VK 3: MS (ESI): *m/z* = 809 $[M+Na]^+$. HRMS (ESI): *m/z* calcd. for $[C_{43}H_{54}FeO_{10}Na]^+$: 809.29536, found: 809.29594. Anal. calcd. for $C_{43}H_{54}FeO_{10}$: C, 65.65; H, 6.92, Found: C, 65.62.17; H, 7.18.

^{1}H-NMR spectrum of hybrid **TF19** recorded on a Bruker Avance spectrometer (300 MHz, $CDCl_3$):

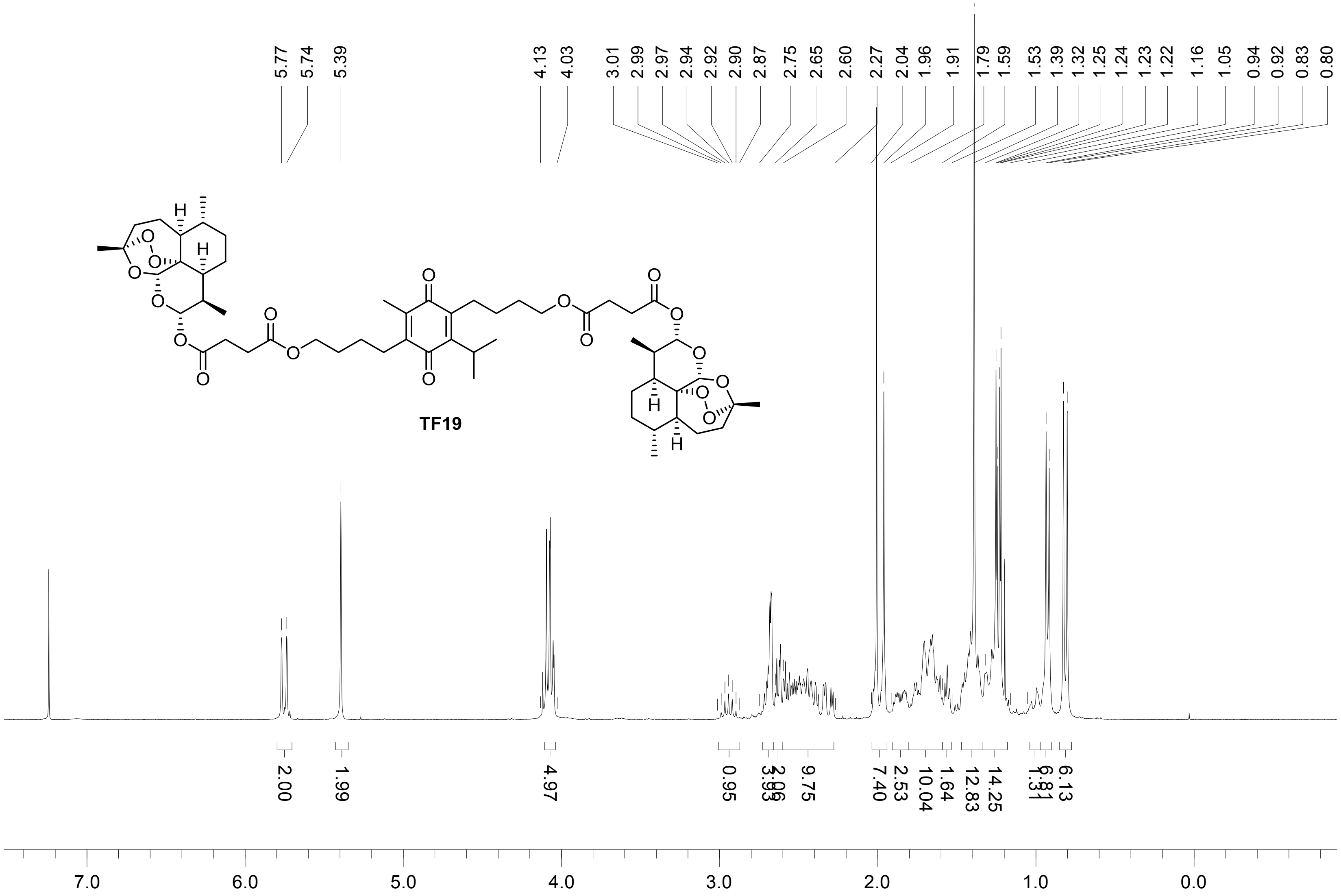

^{13}C-NMR spectrum of hybrid **TF19** recorded on a Bruker Avance spectrometer (75 MHz, $CDCl_3$):

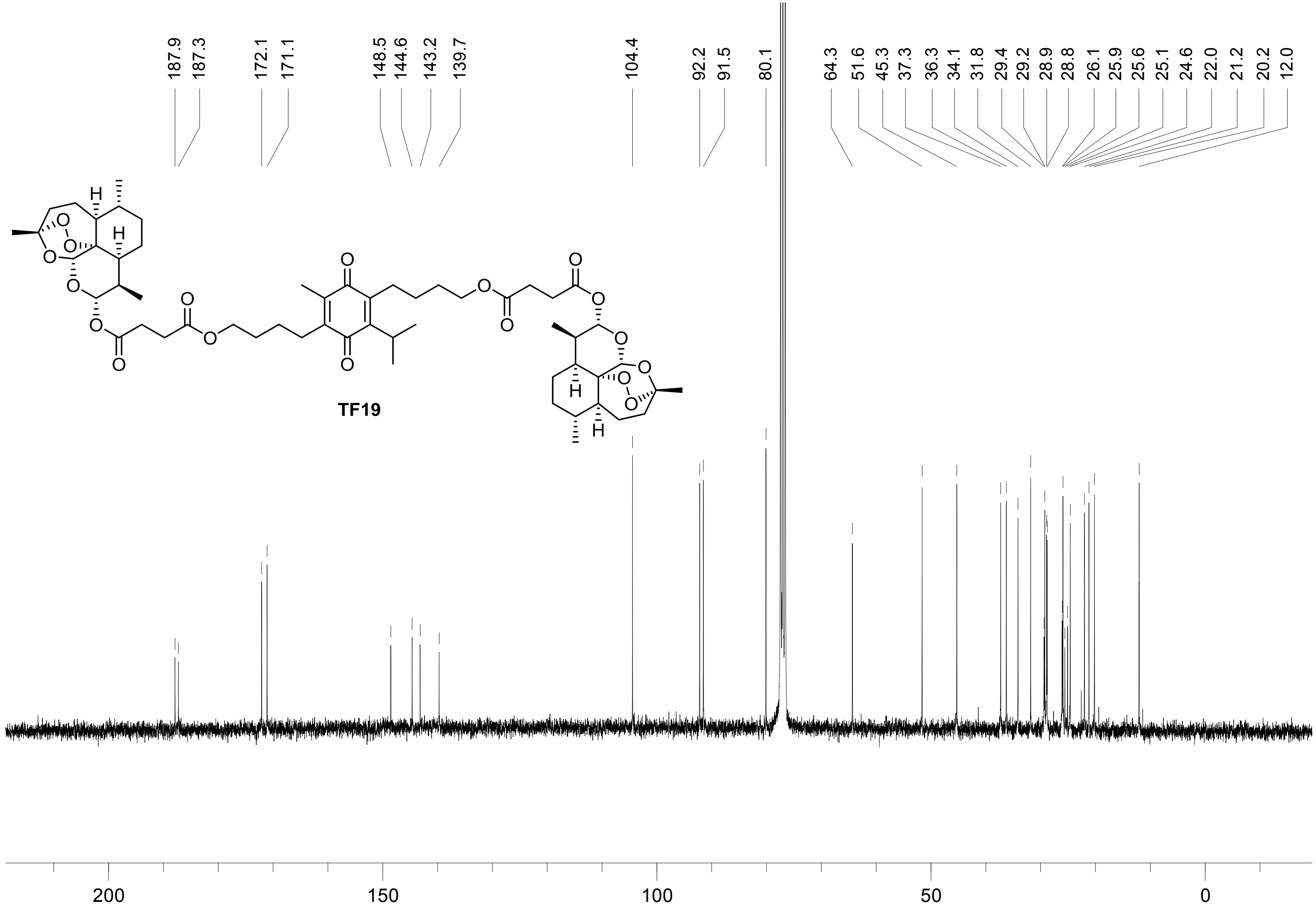

^{1}H-NMR spectrum of hybrid **TF26** recorded on a Bruker Avance spectrometer (300 MHz, $CDCl_3$):

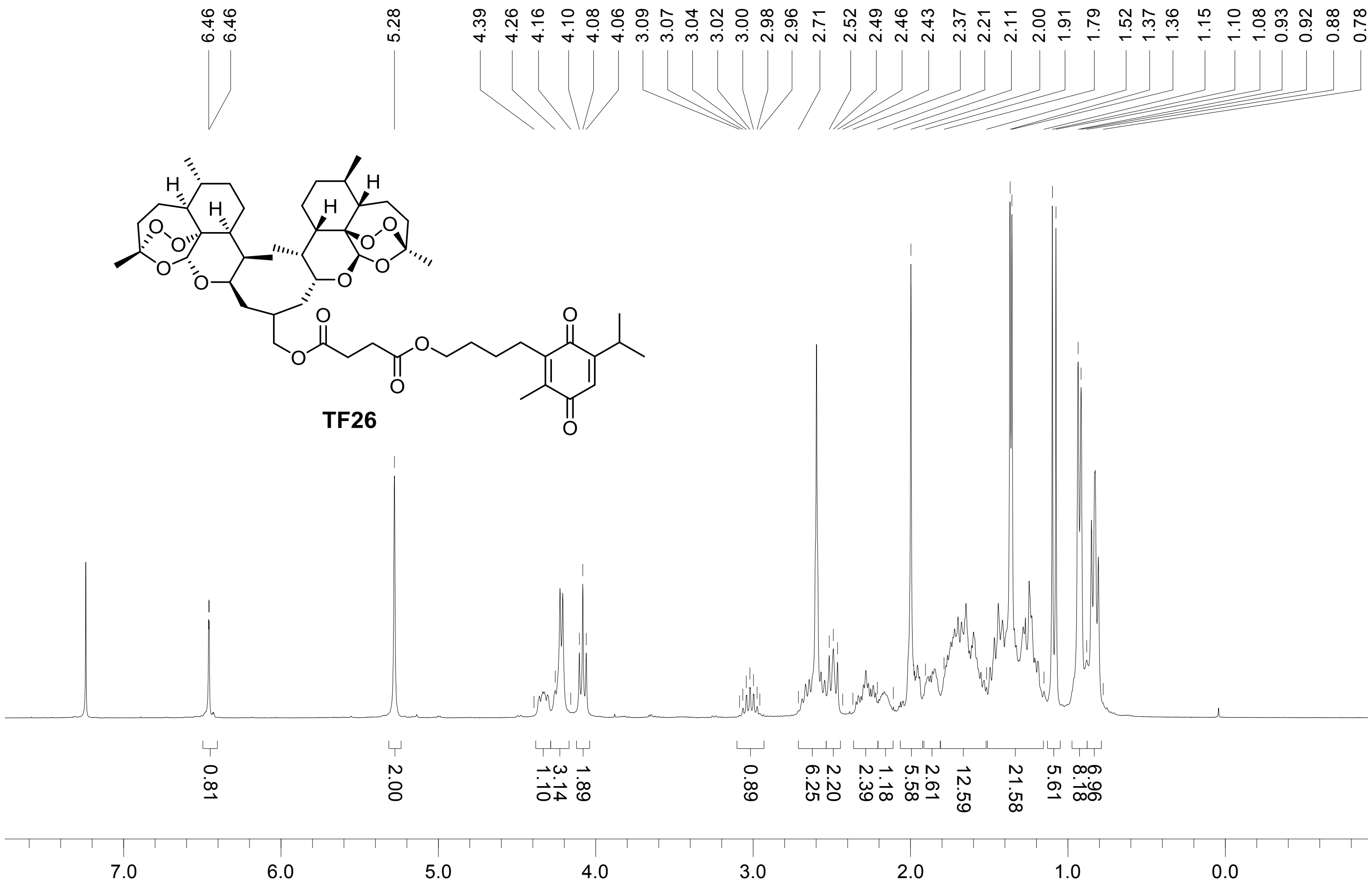

^{13}C-NMR spectrum of hybrid **TF26** recorded on a Bruker Avance spectrometer (75 MHz, $CDCl_3$):

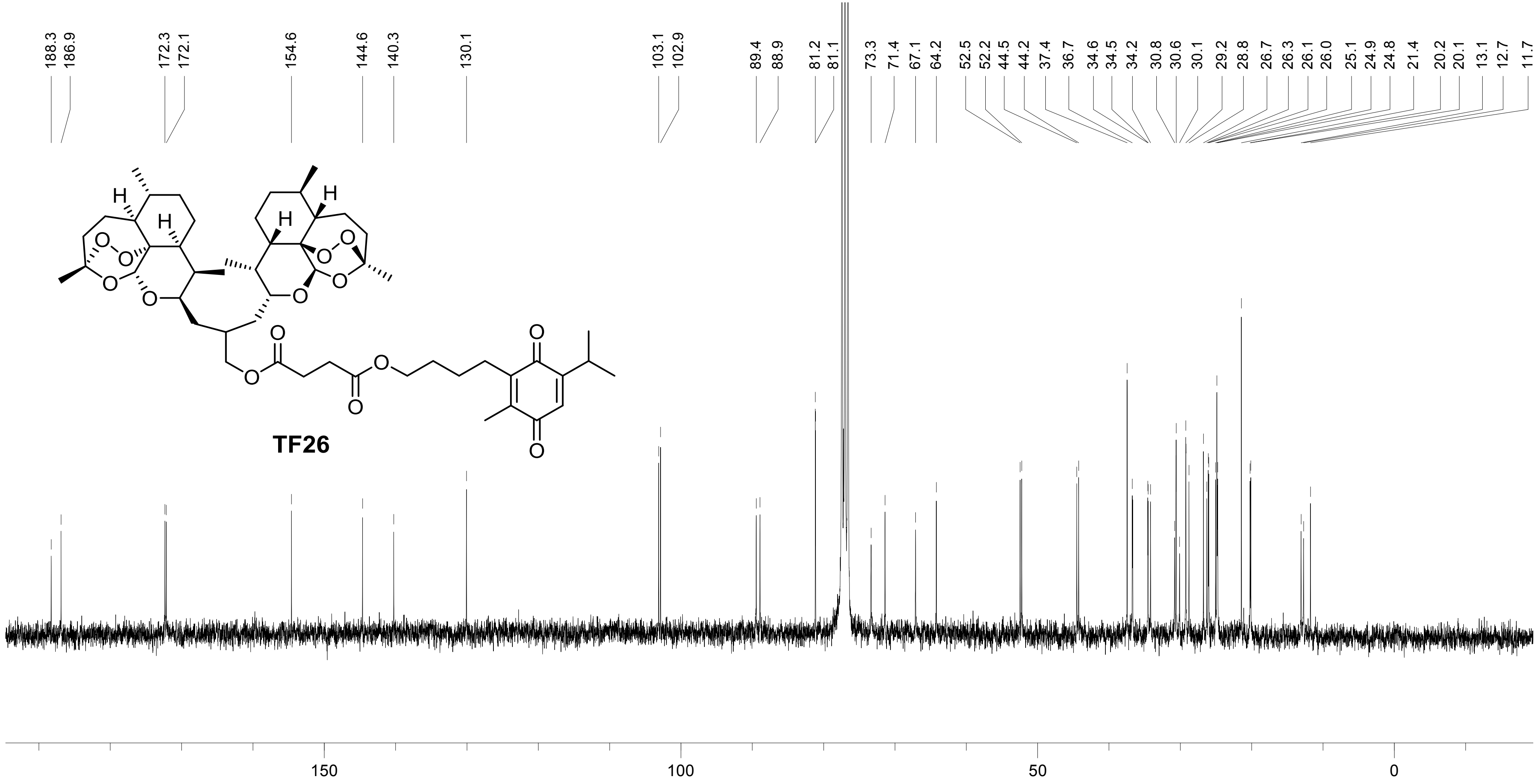

^{1}H-NMR spectrum of hybrid **REI 213** recorded on a Bruker Avance spectrometer (300 MHz, $CDCl_3$):

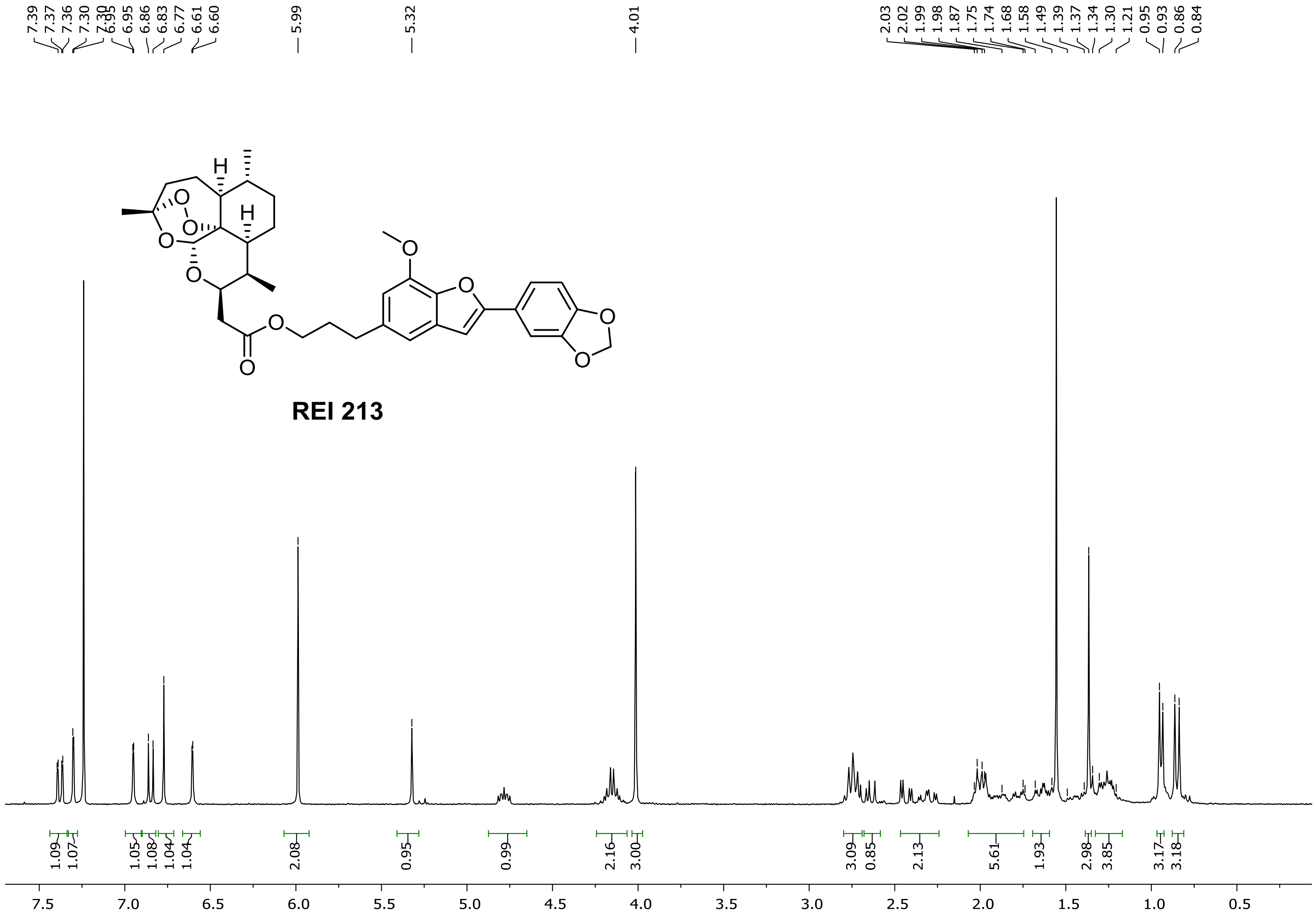

^{13}C-NMR spectrum of hybrid **REI 213** recorded on a Bruker Avance spectrometer (75 MHz, $CDCl_3$):

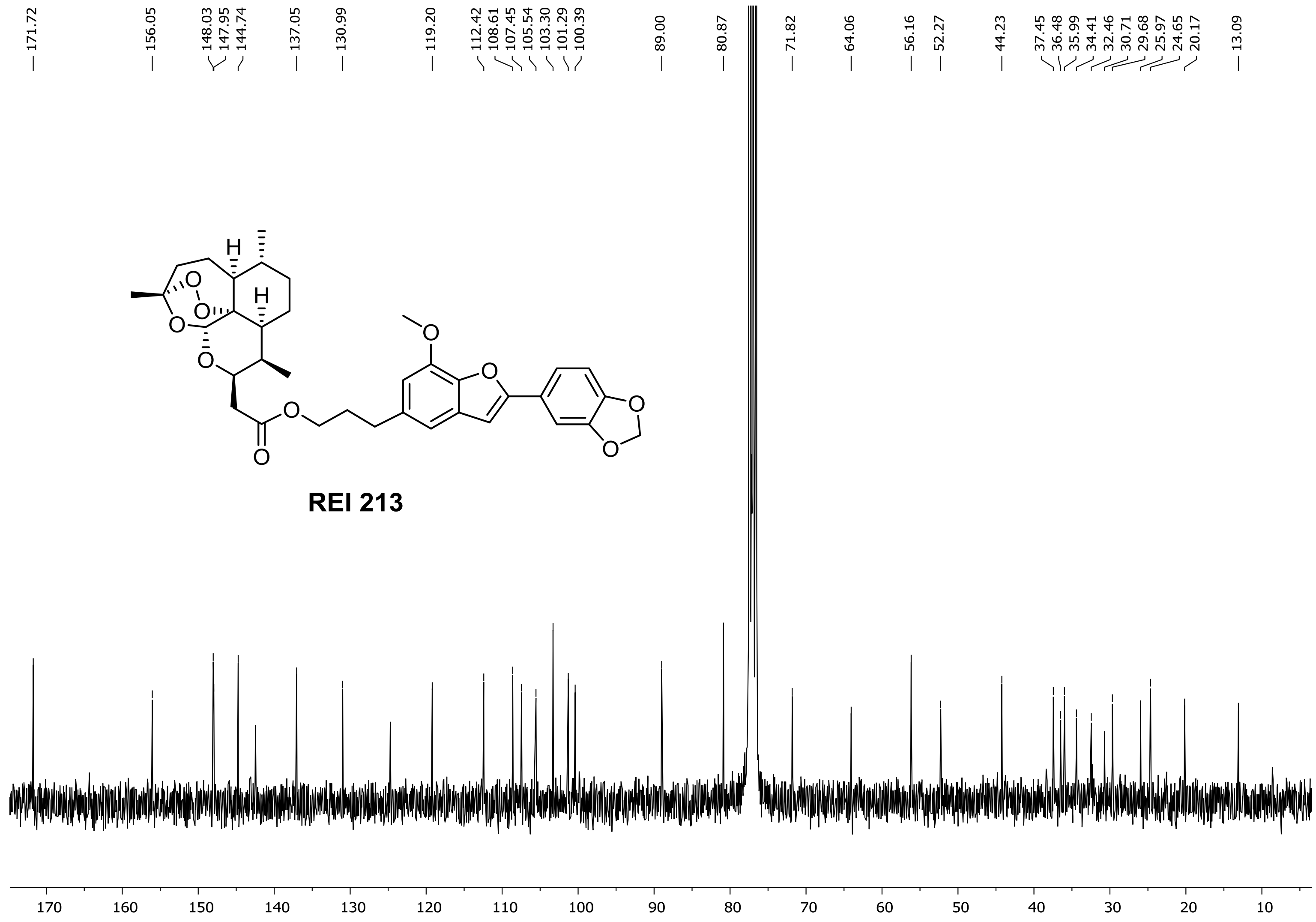

^{1}H-NMR spectrum of hybrid **REI 220+216** recorded on a Bruker Avance spectrometer (300 MHz, $CDCl_3$):

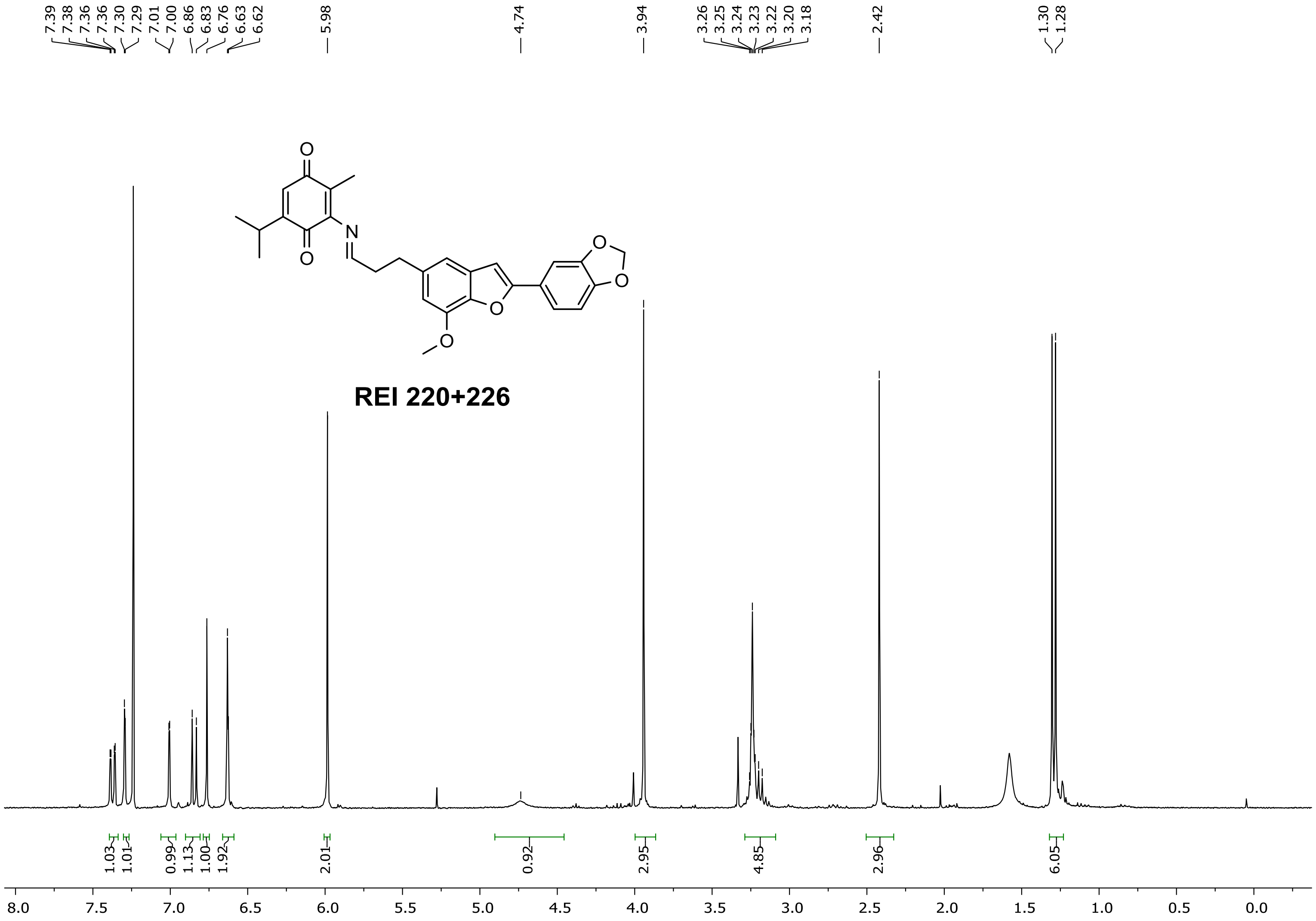

^{13}C-NMR spectrum of hybrid **REI 220+216** recorded on a Bruker Avance spectrometer (75 MHz, $CDCl_3$):

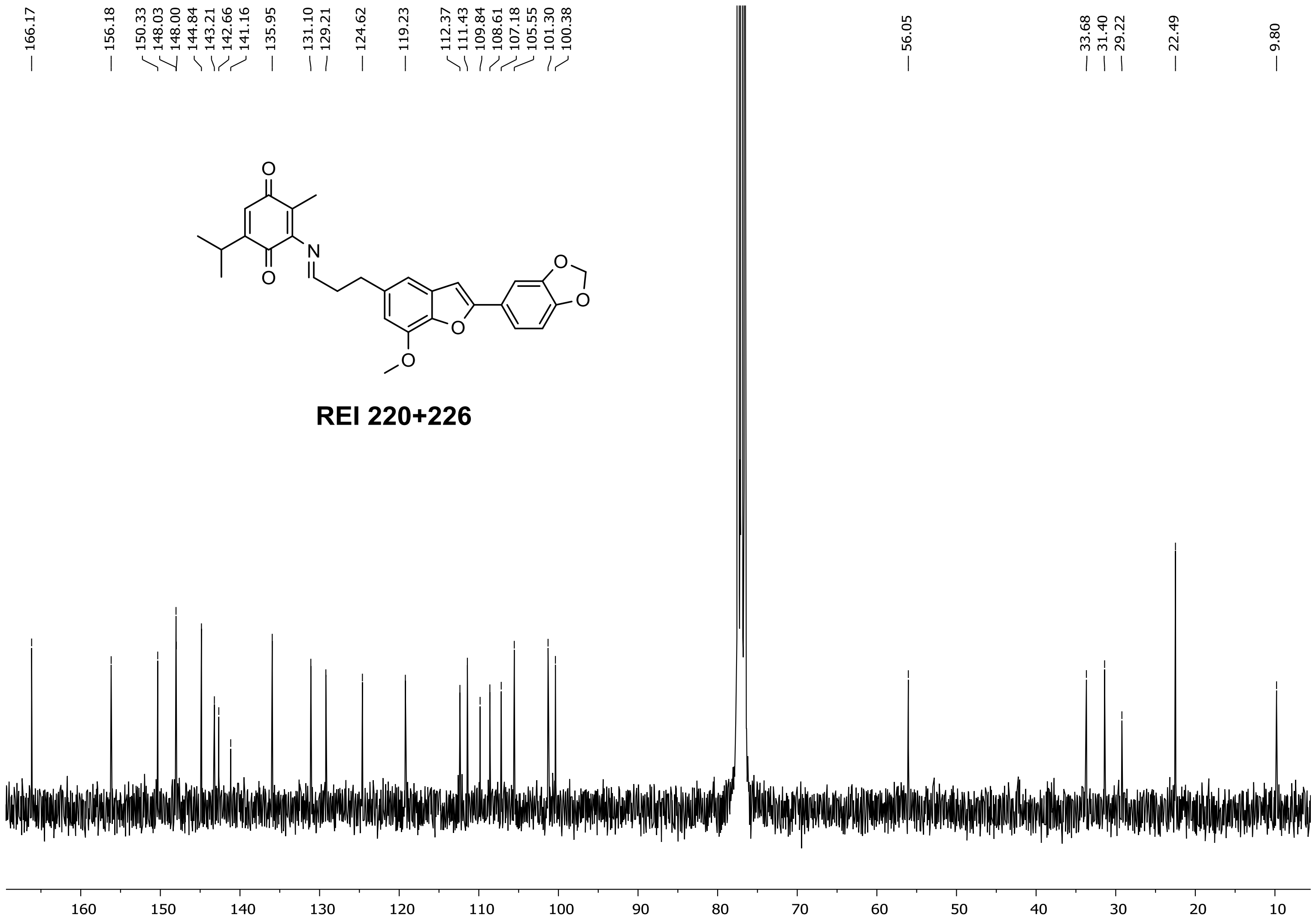

^{1}H-NMR spectrum of hybrid **REI 234** recorded on a Bruker Avance spectrometer (300 MHz, $CDCl_3$):

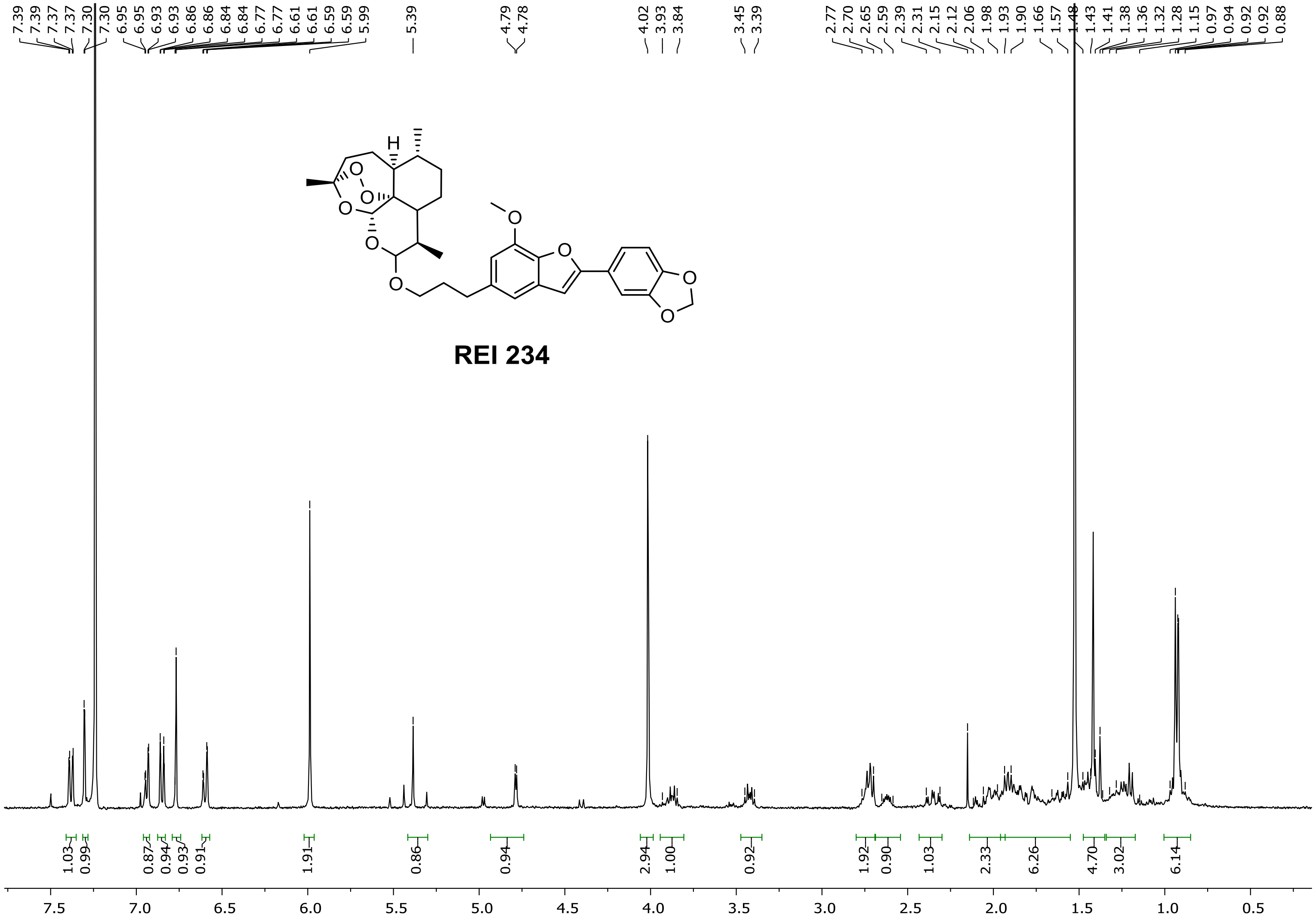

^{13}C-NMR spectrum of hybrid **REI 234** recorded on a JEOL JNM GX 400 spectrometer (100 MHz, $CDCl_3$):

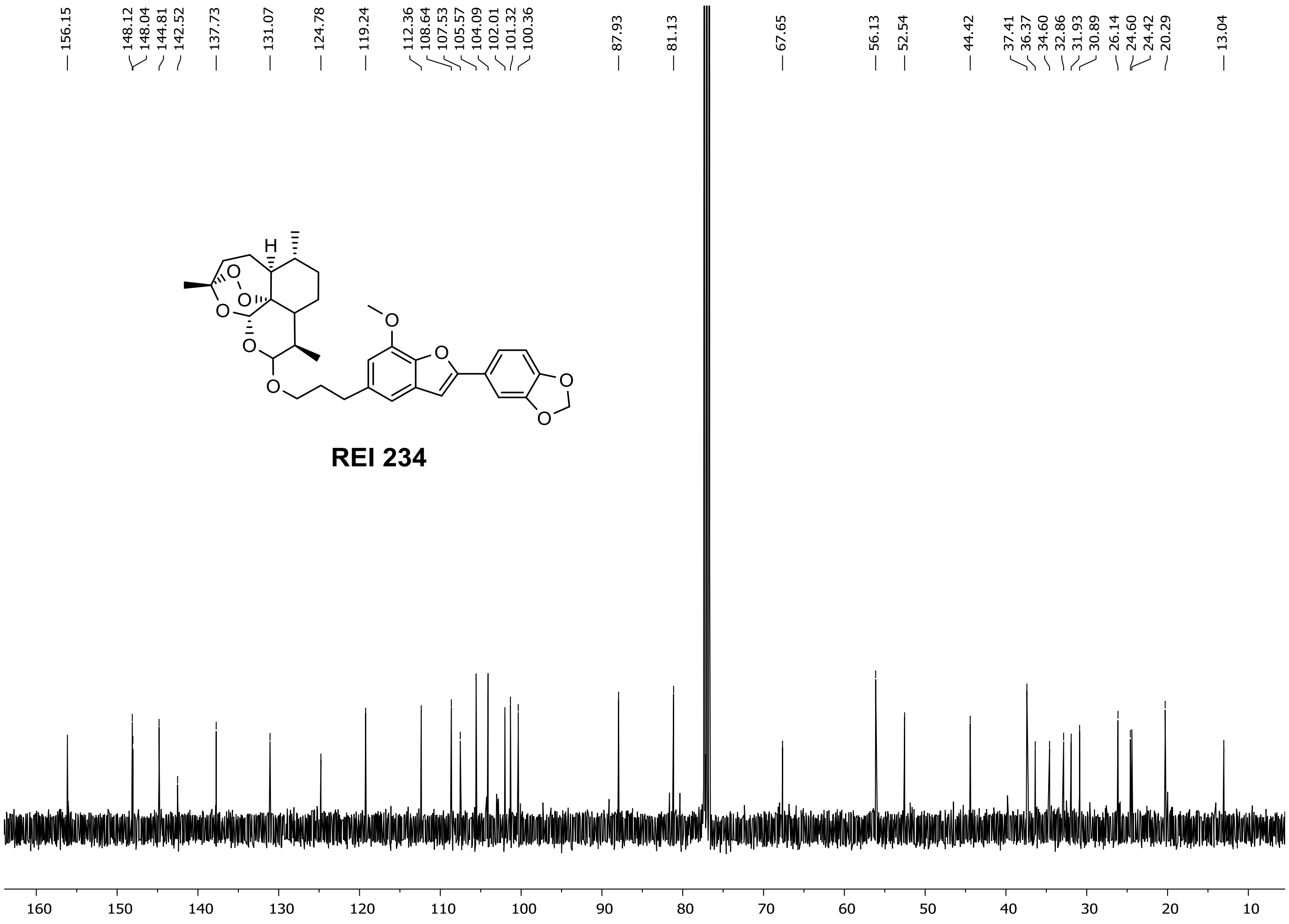

^{1}H-NMR spectrum of hybrid **REI 259** recorded on a JEOL JNM GX 400 spectrometer (400 MHz, Acetone-d_6):

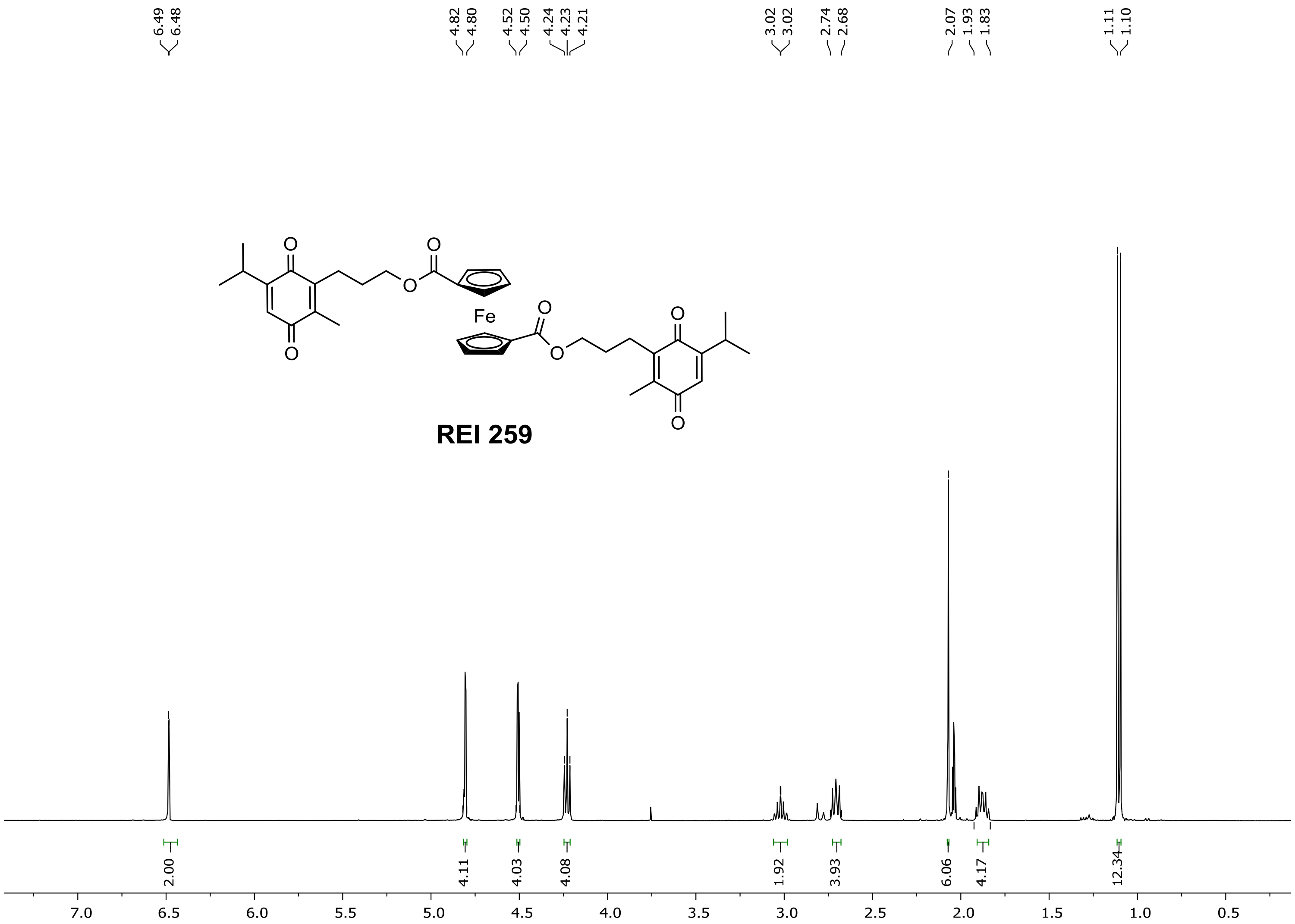

^{13}C-NMR spectrum of hybrid **REI 259** recorded on a JEOL JNM GX 400 spectrometer (100 MHz, Acetone-d_6):

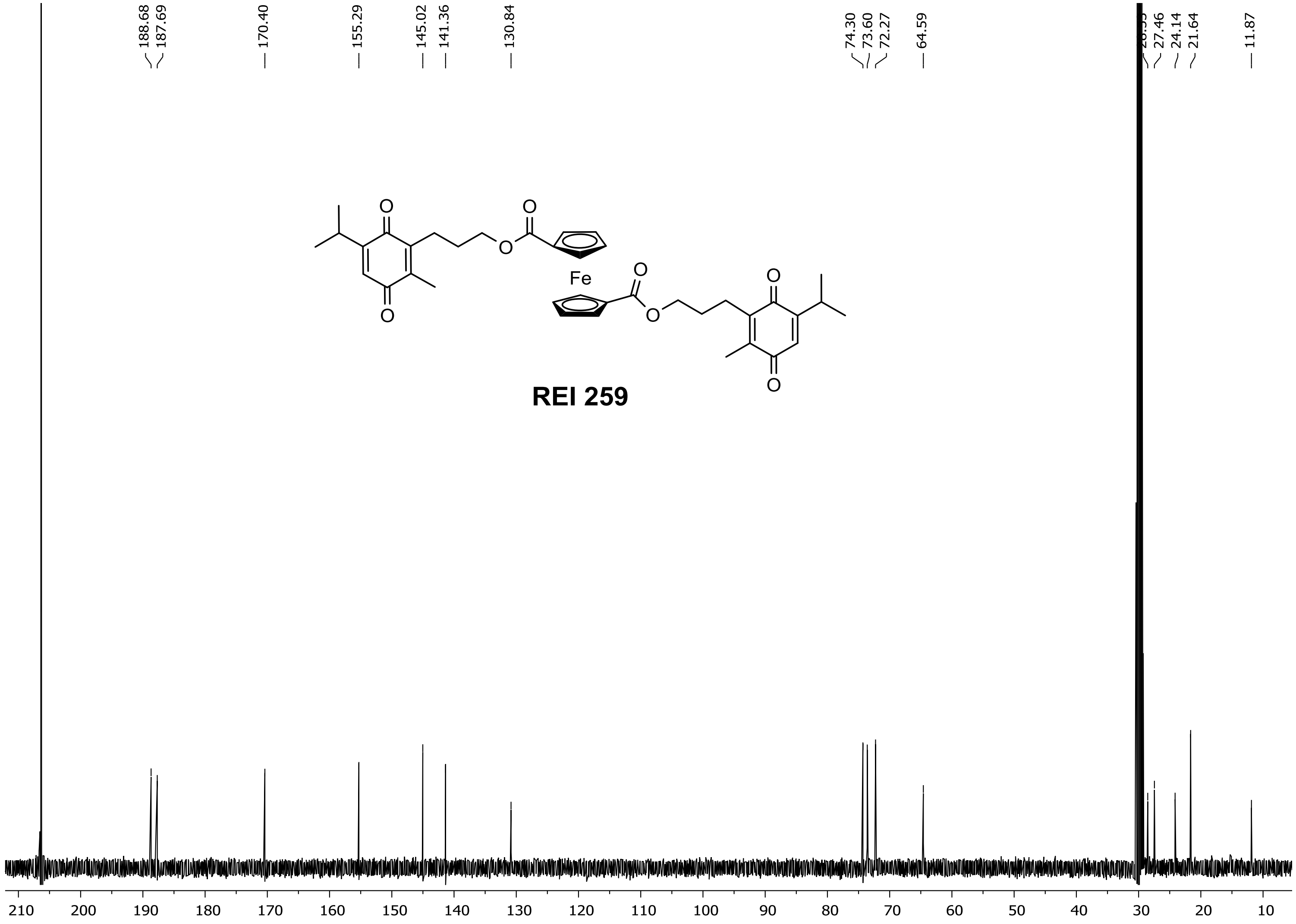

^{1}H-NMR spectrum of hybrid **HH3** recorded on a Bruker Avance spectrometer (300 MHz, Acetone-d_6):

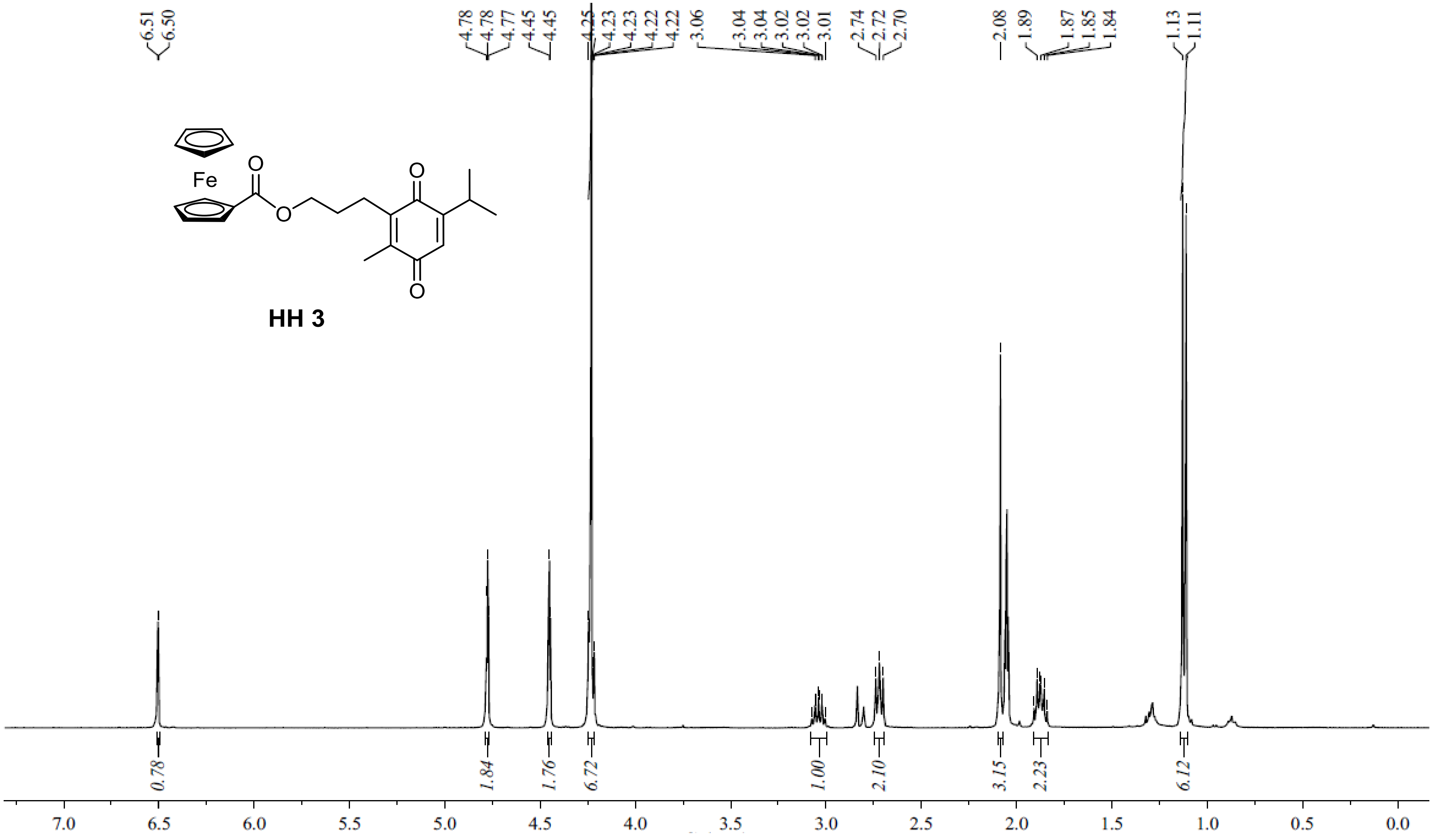

^{13}C-NMR spectrum of hybrid **HH3** recorded on a Bruker Avance spectrometer (75 MHz, Acetone-d_6):

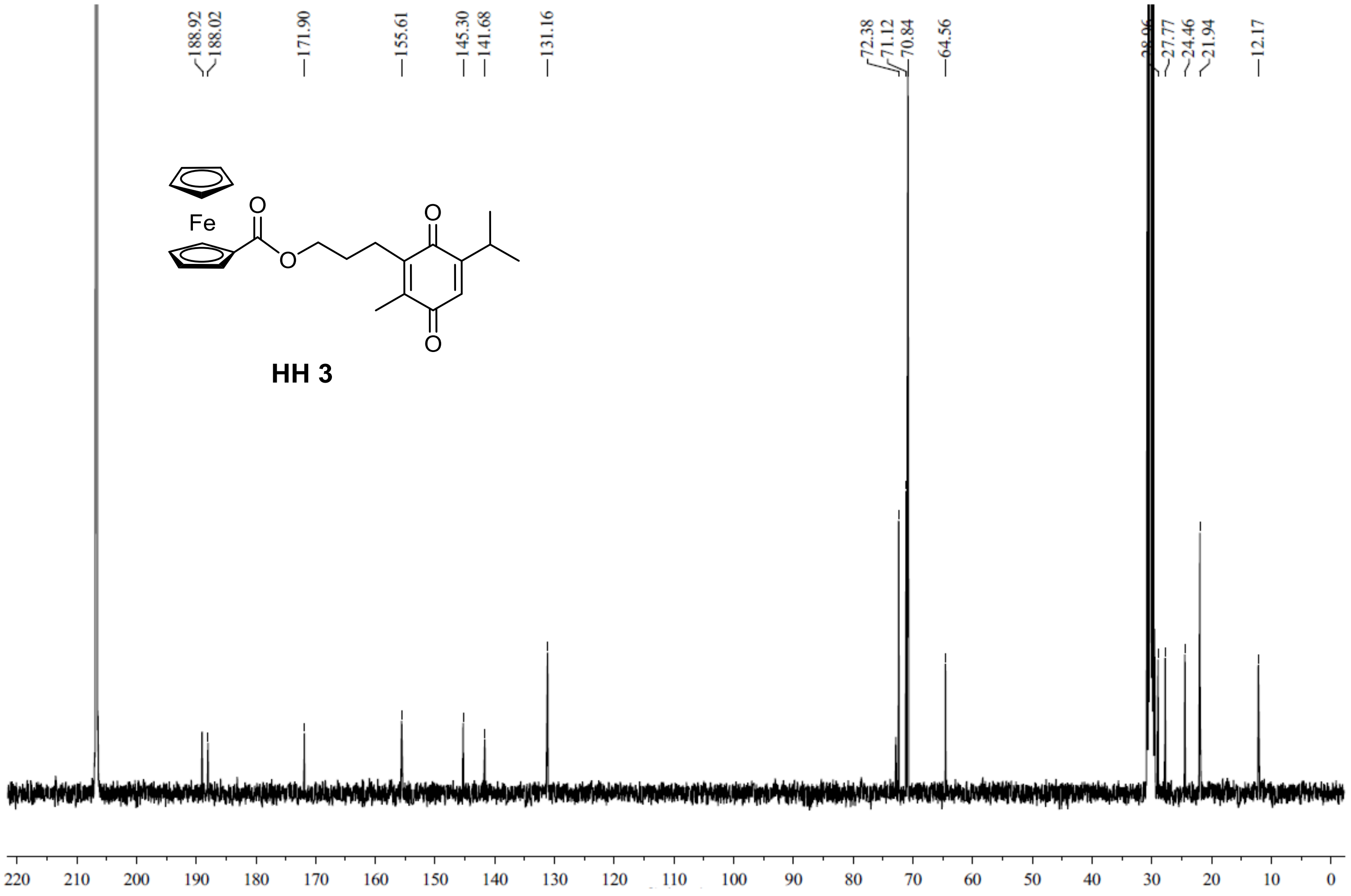

^{1}H-NMR spectrum of hybrid **VK3** recorded on a JEOL JNM GX 400 spectrometer (400 MHz, Acetone-d_6):

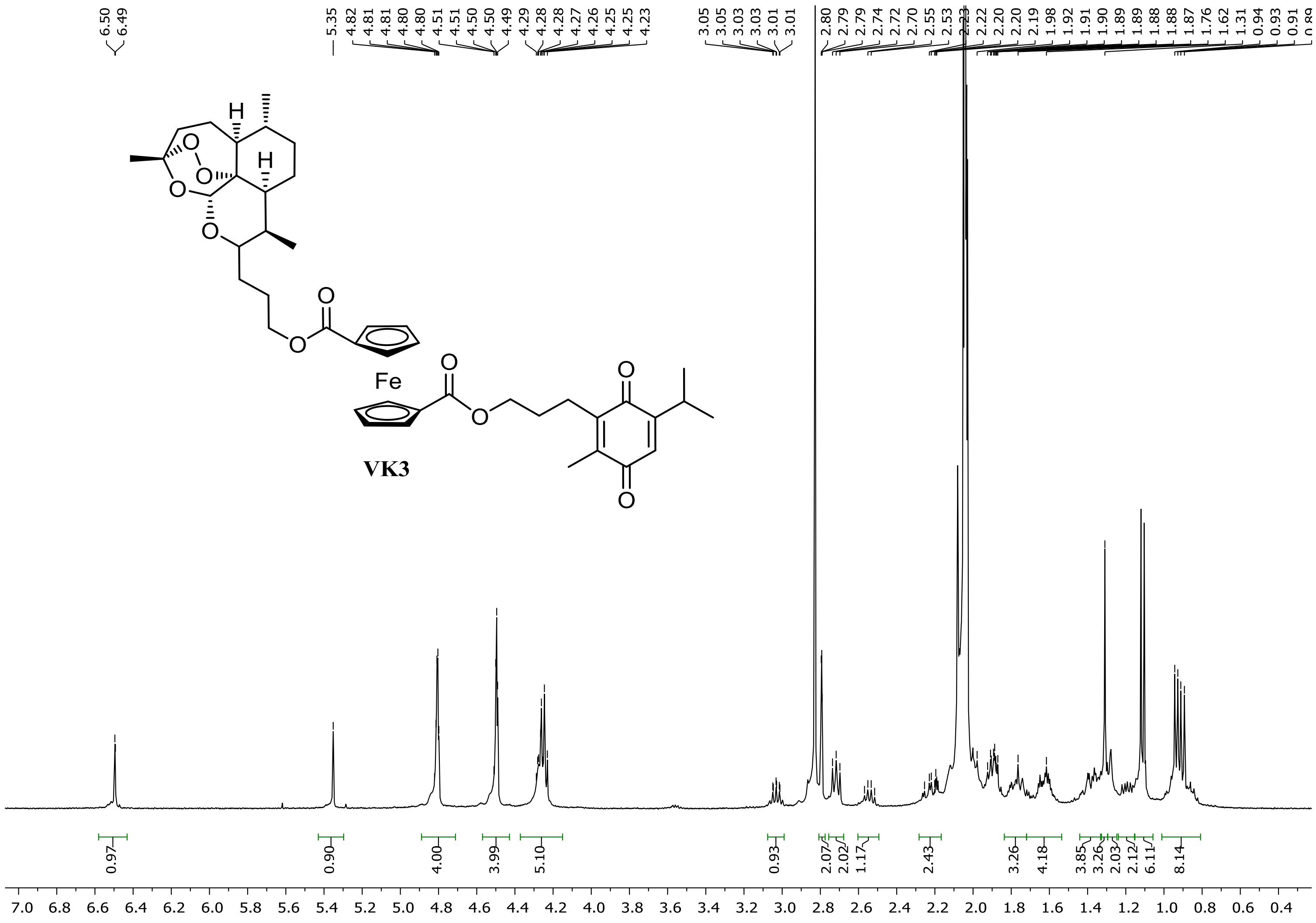

^{13}C-NMR spectrum of hybrid **VK3** recorded on a JEOL JNM GX 400 spectrometer (100 MHz, Acetone-d$_6$):

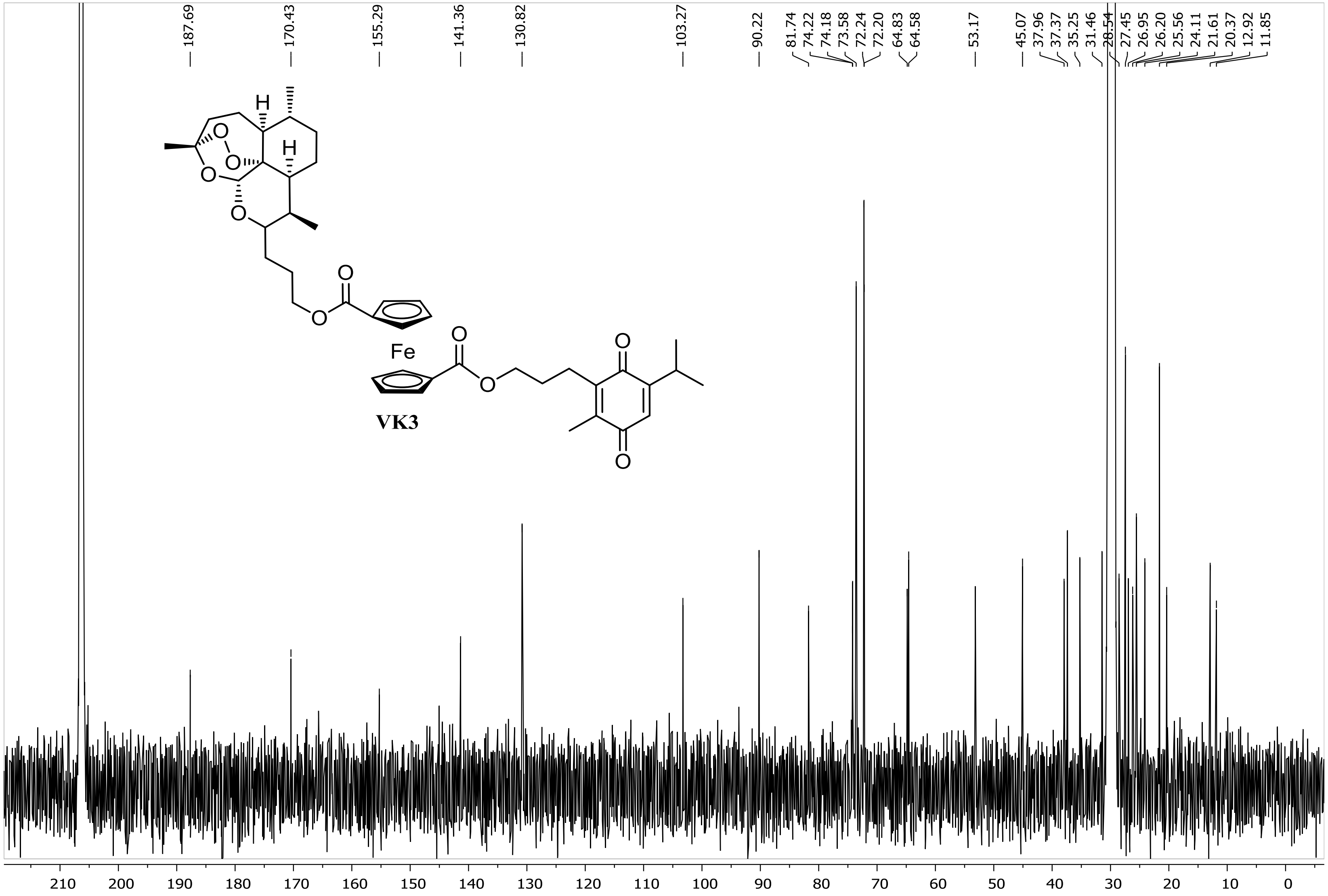

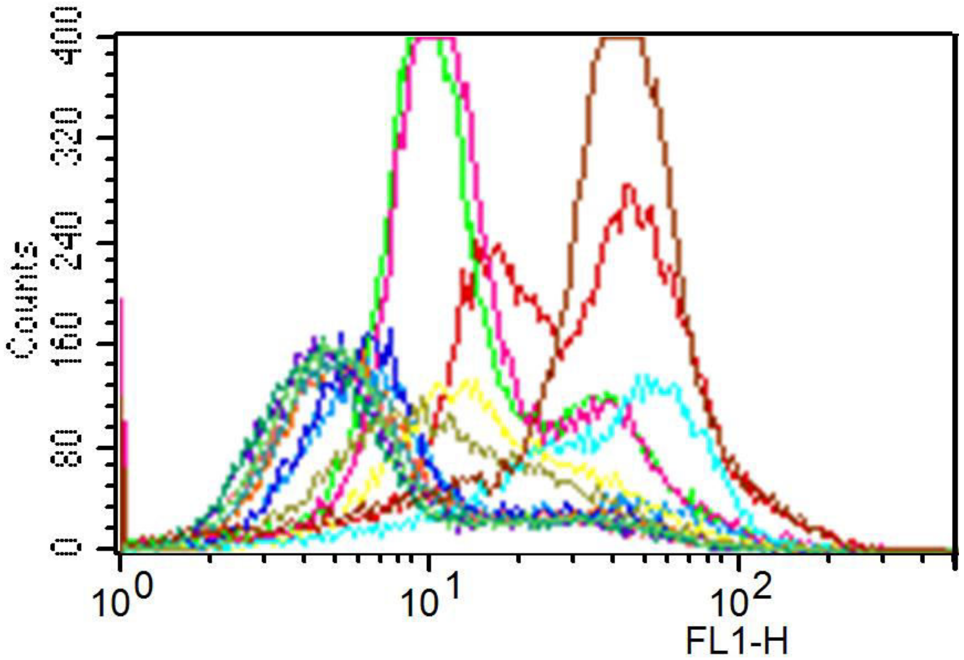

Purple	Doxorubicin alone
Dark green	DOX + TF26
Orange	DOX + REI213
Light blue	DOX + REI220+26
Rosy	DOX + VK3
Dark blue	DOX + TF27
Grey	DOX + REI230
Middle blue	DOX + HH3
Dark brown	DOX + REI234
Pink	DOX + DHA
Light green	DOX + TF29
Yellow	DOX + TF19
Cyan blue	DOX + REI259
Red	DOX + REI235
Brown	DOX + Verapamil

Figure S2: Representative flow cytometric histograms of doxorubicin uptake in CEM/ADR5000 cells. Cells were incubated for 24 h with doxorubicin alone (purple curve on the left), doxorubicin (DOX) plus ART derivatives (from left to right: TF26, REI213, REI220+26, VK3, TF27, REI230, HH3, REI234, DHA, TF29, TF19, REI259, REI235), or doxorubicin plus verapamil (brown curve on the right. Shown are the fluorescence intensities (x-axis) and cell counts (y-axis).
